# Prognostic role of STAT3 in solid tumors: a systematic review and meta-analysis

**DOI:** 10.18632/oncotarget.7887

**Published:** 2016-03-03

**Authors:** Pin Wu, Dang Wu, Lufeng Zhao, Lijian Huang, Gang Shen, Jian Huang, Ying Chai

**Affiliations:** ^1^ Department of Thoracic Surgery, Second Affiliated Hospital, Zhejiang University School of Medicine, Zhejiang University, Hangzhou, 310009, China; ^2^ Department of Radiation Oncology, Second Affiliated Hospital, Zhejiang University School of Medicine, Zhejiang University, Hangzhou, 310009, China; ^3^ Department of Surgical Oncology, Second Affiliated Hospital, Zhejiang University School of Medicine, Zhejiang University, Hangzhou, 310009, China; ^4^ Cancer Institute, Second Affiliated Hospital, Zhejiang University School of Medicine, Zhejiang University, Hangzhou, 310009, China

**Keywords:** STAT3, solid tumors, prognosis, overall survival, disease free survival

## Abstract

Accumulated studies have provided controversial evidences of the association between signal transducer and activator of transcription proteins 3 (STAT3) expression and survival of human solid tumors. To address this inconsistency, we performed a meta-analysis with 63 studies identified from PubMed, Medline and EBSCO. We found STAT3 overexpression was significantly associated with worse 3-year overall survival (OS) (OR = 2.06, 95% CI = 1.57 to 2.71, *P* < 0.00001) and 5-year OS (OR = 2.00, 95% CI = 1.53 to 2.63, *P* < 0.00001) of human solid tumors. Similar results were observed when disease free survival (DFS) were analyzed. Subgroup analysis showed that elevated STAT3 expression was associated with poor prognosis of gastric cancer, lung cancer, gliomas, hepatic cancer, osteosarcoma, prostate cancer, pancreatic cancer but better prognosis of breast cancer. The correlation between STAT3 and survival of solid tumors was related to its phosphorylated state. High expression level of STAT3 was also associated with advanced tumor stage. In conclusion, elevated STAT3 expression is associated with poor survival in most solid tumors. STAT3 is a valuable biomarker for prognosis prediction and a promising therapeutic target in human solid tumors.

## INTRODUCTION

Signal transducer and activator of transcription proteins 3 (STAT3) is well demonstrated to play a crucial role in tumor development and cancer-related inflammation [1]. STAT3 is also linked to inflammation-related oncogenesis initiated by genetic alterations and environmental factors [2–4], and is constitutively activated in various cancers [5, 6]. Persistent activation of STAT3 is involved in promoting tumor cell proliferation, survival, tumor invasion, angiogenesis and immunosuppression, inducing and maintaining a pro-carcinogenic inflammatory microenvironment [7]. Growing studies identified novel tumor-promoting functions of STAT3 in mitochondria metabolism [8], drug resistance [9, 10], epigenetic regulation [11], cancer stem cells [12, 13] and pre-metastatic niches [14, 15]. Given the pivotal role in tumor development, STAT3 represents an attractive therapeutic target for solid tumors. Recently, accumulating studies have demonstrated STAT3-targeted therapy could effectively restrain tumor development in various solid tumors [16–21]. However, the prognostic value of STAT3 overexpression in human solid tumors is still controversial.

A plenty of studies showed that elevated STAT3 expression in tumor tissue was correlated with poor survival of patients with various solid tumors such as gastric cancer [22–29], lung cancer [30–37], gliomas [38–42], colorectal cancer [43], ovarian cancer [44], cervical cancer [45], hepatocellular carcinoma [46, 47], melanoma [48], esophageal cancer [49], osteosarcoma [50, 51], pancreatic cancer [52, 53], thymic epithelial tumor [54], astrocytomas [55], lingual squamous cell carcinoma [56], nasopharyngeal carcinoma [57], prostate cancer [58], renal cell carcinoma [59] and Wilms' tumor [60]. However, other studies reported that overexpression of STAT3 was correlated with favorable outcome of patients with breast cancer [61–65], gastric cancer [66], lung cancer [67–69], colorectal cancer [70, 71] and melanoma [72].

Therefore, we carried out a meta-analysis combining available evidences to evaluate the prognostic value of STAT3 expression in solid tumors. We also evaluated whether the clinical outcome of patients with solid tumors differed between STAT3 phosphorylation state and between different tumor types. This meta-analysis intended to assess the role of STAT3 in relation to survival in solid tumors, thereby supporting more rational development of therapeutic strategies against STAT3.

## RESULTS

### Search results and study characteristics

**Figure 1:**
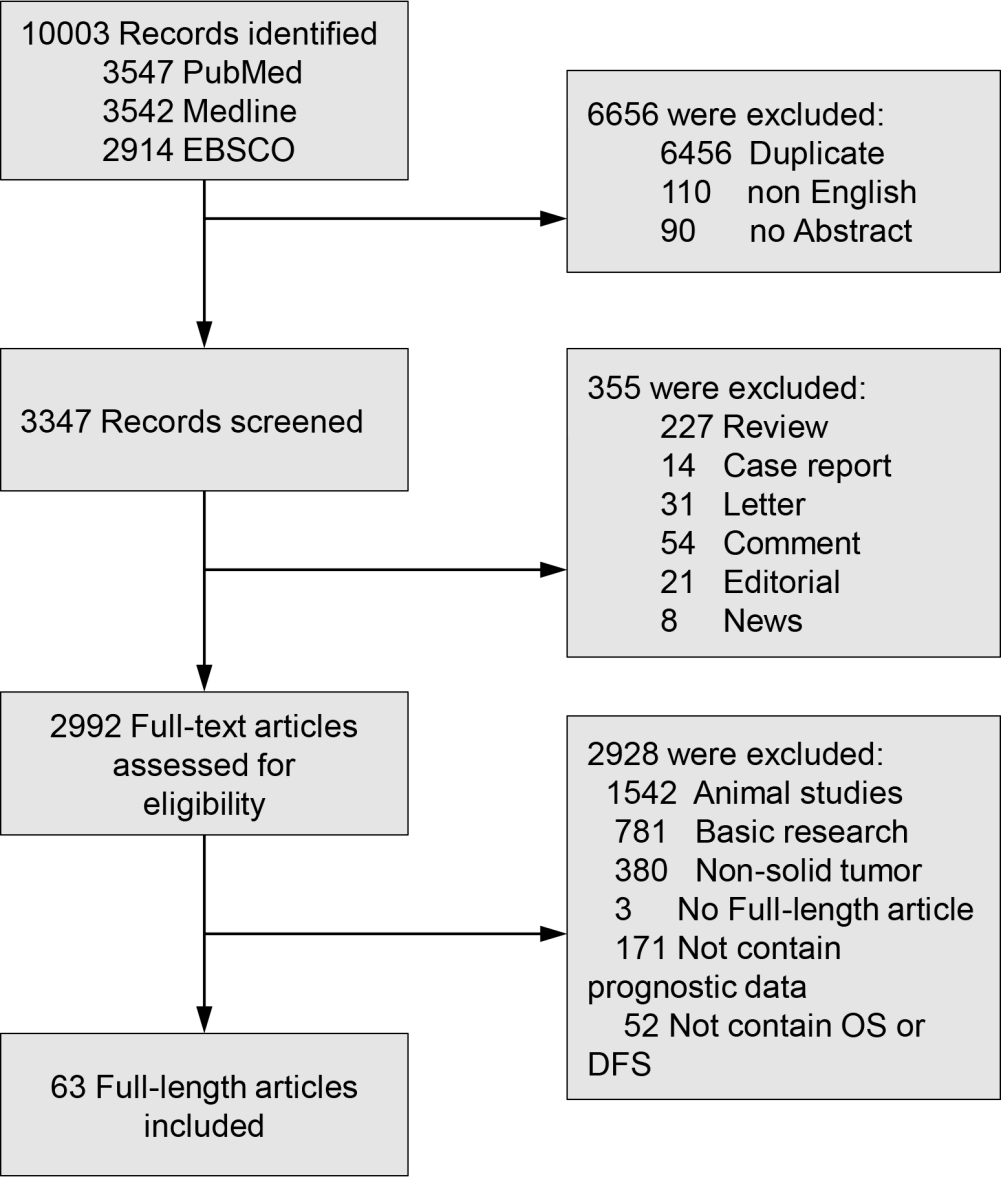
Flow diagram of study selection STAT3: Signal transducer and activator of transcription protein 3; OS: overall survival; DFS: disease-free survival.

Sixty-three studies with a total of 9449 patients were included (Figure 1). Characteristics of included studies are shown in Table 1. Eleven studies evaluated lung cancer [30–37, 67–69], nine evaluated gastric cancer [22–29, 66], five evaluated breast cancer [61–65], five evaluated gliomas [38–42], four evaluated colorectal cancer [43, 70, 71, 73], three evaluated ovarian cancer [44, 74, 75], three evaluated cervical cancer [45, 76, 77], two evaluated hepatocellular carcinoma [46, 47], two evaluated melanoma [48, 72], two evaluated esophageal cancer [49, 78], two evaluated osteosarcoma [50, 51], two evaluated pancreatic cancer [52, 53], two evaluated thymic epithelial tumours [54, 79], two oral cancer [80, 81], and one each evaluated astrocytomas [55], chordoma [82], head and neck squamous cell carcinoma [83], lingual squamous cell carcinoma [56], nasopharyngeal carcinoma [84], pharyngeal cancer [57], prostate cancer [58], renal cell carcinoma [59], and Wilms' tumor [60]. Of these 63 studies, 20 studies evaluated STAT3, 37 studies evaluated p-STAT3, and 6 studies evaluated both STAT3 and p-STAT3. As for the region, 39 studies were conducted in Asia, 13 studies in America, 10 studies in Europe, and 1 study in Austria.

**Table 1:** Characteristics of studies included in the meta-analysis

References	Country	Type of cancer	Patient No.	Age, median (range)	Male/Female	Stage	Follow-Up,months (Range)	STAT3 (+/–) NO.	3-yearOS (+/–)%	5-yearOS (+/–)%	NOS Score
**Studies including OS**											
Abou-Ghazal, M., et al. (2008)	USA	Gliomas	128	44 (4–91)	NR	II-IV	NR	65/63	36.7/48.9	34.7/41.7	7
Ai, T., et al. (2012)	China	NSCLC	65	NR	50/15	I-IV	29.9 ± 15.7	47/18	49.8/77.9	NR	8
Birner, P., etal. (2010)	Bulgaria	Gliomas	111	58.0 ± 11.6	57/54	NR	11.1 ± 0.8	65/46	0/17.9	NR	7
Chang, K. C., et al. (2006)	China	TET	118	52.7 (25–77)	65/53	I-IV	NR	38/80	58.7/56.7	32.9/30	7
Chatterjee, Devasis., et al. (2008)	USA	GC	143	71.1 (31–96)	75/68	IA-IV	34 (12–180)	40/103	45.1/70.1	0/60.7	8
Chen, C. C., et al. (2010)	China	NC	95	NR	NR	I-IV	112.8 (31.2–240)	34/61	39/61.2	31.3/53	6
Chen, H. H., et al. (2012)	China	CC	165	69 (29–89)	0/165	I-IV	NR	36/129	53.8/60	47.3/48.5	8
Cortas, T., et al. (2007)	USA	NSCLC	145	70 (40–88)	64/81	I-III	35 (4–85)	50/84	70/60.7	55.5/52	7
Deng, J. Y., et al. (2010)	China	GC	53	55 (31–78)	37/16	I-IV	38 (2–108)	26/27	11.5/85.2	3.8/85.2	8
Deng, J., et al. (2013)	China	GC	114	NR	76/38	NR	NR	89/25	24.6/80	9.9/50.6	7
Denley, S. M., et al. (2013)- Tyr705	UK	PDA	86	NR	43/43	NR	22	29/57	9/21.9	0/11	7
Denley, S. M., et al. (2013)- Ser727	UK	PDA	86	NR	43/43	NR	22	30/56	83/22.2	0/11.2	7
Dolled-F. M., et al. (2003)-M1-C	USA	BC	286	NR	0/286	NR	NR	198/88	94.6/87.3	85.5/80	6
Dolled-F. M., et al. (2003)-M1-N	USA	BC	286	NR	0/286	NR	NR	66/220	94.6/92.1	93.1/81.2	6
Dolled-F. M., et al. (2003)-M2-C	USA	BC	286	NR	0/286	NR	NR	56/229	92.5/92.5	86.7/83.7	6
Dolled-F. M., et al. (2003)-M2-N	USA	BC	286	NR	0/286	NR	NR	124/161	95.7/89.9	91.3/78.7	6
Galleges Ruiz, M. I., et al. (2009)	USA	NSCLC	178	NR	127/51	I-III	NR	51/111	54.5/45	48.8/36.5	7
Gordziel, C., et al. (2013)-C	Germany	CRC	414	NR	NR	I-III	37 (0–146)	132/282	82.8/70.6	74/61	6
Gordziel, C., et al. (2013)-N	Germany	CRC	414	NR	NR	I-III	37 (0–146)	124/290	78.6/72	71.2/62.1	6
Haura, Eric B., et al. (2005)	USA	NSCLC	176	69 (45–84)	97/79	I	72 (36–108)	94/82	77.4/74.3	57.8/52.2	8
Hbibi, A. Tadlaoui., et al. (2008)-M1	France	CRC	126	68.1	NR	I-IV	NR	62/38	NR	61.3/49.2	7
Hbibi, A. Tadlaoui., et al. (2008)-M2	France	CRC	126	68.1	NR	I-IV	NR	27/73	NR	59.1/55.6	7
Horiguchi, Akio., et al. (2002)	Japan	RCC	48	63 (24–85)	39/9	I-IV	15.9 (1–101	24/24	53.5/89.7	53.5/89.7	7
Huang, C., et al. (2012)	China	PDA	71	67 (40–80)	50/21	I-IV	33.7 (3–60)	39/32	0/24.3	0/14.2	8
Jia, Yanfei., et al. (2013)	China	GC	48	66 (45–83)	34/14	I-IV	NR	19/29	54.4/86.7	12.7/47.6	7
Kim, D. Y., et al. (2009)	Korea	GC	71	NR	48/23	I-IV	30 (11–83)	27/44	59/86.4	40/81.8	6
Kim, Yeon-Joo., et al. (2011)	Korea	NC	38	48 (25–74)	30/8	I-IV	43.7 (0.71–60)	10/28	NR	41/77	8
Kusaba, T., et al. (2006)	Japan	CRC	108	65.6 (44–86)	66/42	I-IV	NR	62/46	61.6/90.1	48.8/90.1	8
Lee, I., et al. (2012)	USA	Melanoma	299	56 (13–85)	212/87	IV	NR	236/63	44.1/41.8	22.3/25.4	7
Lee, J., et al. (2009)	China	GC	303	NR	206/97	II-III	61.5 (12–134)	79/224	68.4/78.4	59.5/70.5	7
Li, Chao., et al. (2013)	China	TET	80	46.5 (19–70)	47/33	I-IV	NR	36/44	70.1/100	46.8/97.6	7
Lin, G. S., et al. (2014)	China	Gliomas	90	55 (18–79).	54/36	NR	46.4 (1.2–109.6)	73/17	14/31.4	NR	8
Mano, Y., et al. (2013)	Japan	HC	101	NR	81/20	NR	NR	36/65	71.3/84.9	60.7/84.7	8
Min, Hao., et al. (2009)-M1	China	OC	50	50.6 (22–73)	0/50	I-IV	NR	44/6	53.5/75.1	29/0	6
Min, Hao., et al. (2009)-M2	China	OC	50	50.6 (22–73)	0/50	I-IV	NR	29/21	35/88.1	0/58.7	6
Monnien, F., et al. (2010)	France	CRC	104	66 (37–80)	76/28	NR	13 (8–48)	39/65	82.2/78	71.8/65.5	7
Pectasides, Eirini., et al. (2010)-1	Greece	HNSC	107	NR	87/20	I-IV	64 (1–120)	23/47	90.4/45.9	72.4/38.3	7
Pectasides, Eirini., et al. (2010)-2	Greece	HNSC	107	NR	87/20	I-IV	64 (1–120)	12/25	NR	68.8/51.4	7
Piperi, Christina., et al. (2011)	Greece	Gliomas	97	59 (19–82)	60/37	II-IV	63 (3–180)	89/8	0/14	NR	6
Rosen, D. G., et al. (2006)	USA	OC	303	58.2 (20–86)	0/303	I-IV	52	215/88	49.9/60	32.1/49.1	8
Ryu, Keinosuke., et al. (2010)	USA	Osteosarcoma	51	20.5 (5–61)	38/13	NR	NR	31/20	48.5/75	35.2/75	8
Schoppmann, S. F., et al. (2012)	Austria	EC	324	63	252/72	NR	NR	144/180	32.6/57.6	24.9/53	7
Sheen-Chen, et al. (2008)	China	BC	102	48.2 (26–76)	0/102	I-III	NR	27/75	NR	59/77.2	6
Slinger, E., et al. (2010)	Sweden	Gliomas	21	NR	NR	NR	NR	7/14	0/13.6	NR	8
Sonnenblick, A., et al. (2012)	Israel	BC	125	NR	0/125	NR	50	35/90	100/91	94.4/76.6	6
Sonnenblick, A., et al. (2013)-1	Israel	BC	375	50	0/375	NR	NR	47/82	97.9/96.3	94/94	7
Sonnenblick, A., et al. (2013)-2	Israel	BC	375	50	0/375	NR	NR	184/150	99/91.9	94.1/80.2	7
Takemoto, S., et al. (2009)	Japan	CC	125	47 (19–77)	0/125	I-II	NR	71/54	80.9/96.8	78.5/94.5	7
Tam, L., et al. (2007)-N	UK	PC	50	70 (64–73)	50/0	NR	29.5 (15–54)	22/28	72.8/92.5	57.9/84.2	8
Tam, L., et al. (2007)-C	UK	PC	50	70 (64–73)	50/0	NR	29.5 (15–54)	19/31	58.1/100	42.5/92.7	8
van Cruijsen, H., et al. (2009)	USA	NSCLC	164	64.5	NR	I-III	NR	116/48	49/58.7	33.1/50.3	7
Wang, M., et al. (2011)	China	NSCLC	208	59.8 (35–76)	NR	I-III	67 (1–78.2)	128/80	53.9/73.2	24.7/39.8	
Wang, Y. C., et al. (2011)	China	Osteosarcoma	76	NR	25/51	NR	37	36/40	25.8/60.4	25.8/60.4	6
Wang, Y., et al. (2011)	China	Gliomas	68	45 (15–68)	41/27	NR	51 (1–72)	47/21	0/15.4	NR	7
Woo, S., et al. (2011)	Korea	GC	285	54.4	193/92	I-IV	39.7 (4–84)	101/179	79/61.6	74.9/54.5	7
Wu, Z.S., et al. (2011)	China	Melanoma	90	NR	52/38	I-IV	NR	51/39	80.5/97.6	50.8/76.7	8
Xiong, Hua., et al. (2012)-M1	China	GC	262	59.3 (23–79)	176/86	I-IV	90 (2–273)	248/14	44/64.3	28.4/42.9	8
Xiong, Hua., et al. (2012)-M2	China	GC	262	59.3 (23–79)	176/86	I-IV	90 (2–273)	136/126	25.3/65.5	11.5/47.3	8
Yakata, Yuichi., et al. (2007)	Japan	GC	111	68.9 (38–89)	63/48	NR	120	55/56	37.7/83.4	37.7/78.6	8
Yamashita, H., et al. (2006)	Japan	BC	506	NR (22–91)	0/506	NR	NR	206/300	92/87.9	86/81.6	7
Yang, C., et al. (2013)	USA	OC	49	61 (41–87)	0/49	I-IV	NR	25/24	70.6/73.4	33.5/57.6	7
Yang, Cao., et al. (2009)	USA	Chordoma	70	59.5 (29–88)	51/19	NR	16.8 (0.8–69.2)	35/35	82.5/90.2	73.1/90.2	8
Yin, Z., et al. (2012)	China	NSCLC	76	NR	48/28	I-IV	NR	42/34	49.6/59.3	41.8/57.9	7
Yu, Y., et al. (2015)	China	NSCLC	82	NR	48/34	I-IV	NR	76/24	28.7/76.2	20.3/48.3	8
Zhang, C. H., et al. (2012)-M1	China	HC	100	55.1 (28–77)	80/20	I-IV	15.4	72/28	53.5/57.8	14/32.2	8
Zhang, C. H., et al. (2012)-M2	China	HC	100	55.1 (28–77)	80/20	I-IV	15.4	58/42	35.5/81.2	19/25.7	8
Zhang, L. J., et al. (2013)	China	Wilms’ tumor	58	31 (3–132)	38/20	I-IV	≥ 78	17/41	45.6/72.1	45.6/72.1	7
Zhao, X., et al. (2012)-M1	China	SCLC	128	NR	66/62	I-IV	67 (1–78.2)	71/57	29.7/81.6	0/9.9	7
Zhao, X., et al. (2012)-M2	China	SCLC	128	NR	66/62	I-IV	67 (1–78.2)	62/66	43.4/62.3	0/3.9	7
Zhao, Yan., et al. (2012)	China	LSCC	163	NR	NR	I-IV	NR	100/63	75/86.7	41.8/78.6	8
**Studies including DFS**											
Choi, Chel Hun., et al. (2010)	Korea	CC	29	NR	0/29	I-II	NR	20/9	49.9/84.6	49.9/84.6	8
Lee, J., et al. (2009)	China	GC	303	NR	206/97	II-III	61.5 (12–134)	79/224	61.7/73.7	58.3/67.7	7
Li, X., et al. (2015)	China	NSCLC	164	NR	115/40	I-III	NR	107/57	57.4/87.6	NR	8
Macha, Muzafar A., et al. (2011)	Canada	Oral cancer	94	NR	70/24	I-IV	NR	63/31	18.6/53.9	7.1/53.9	7
Mano, Y., et al. (2013)	Japan	HC	101	NR	81/20	NR	NR	36/65	12.5/55.6	12.5/31.5	8
Schoppmann, S. F., et al. (2012)	Austria	EC	324	63	252/72	NR	NR	144/180	25.8/48.3	20.2/47.2	7
Takemoto, S., et al. (2009)	Japan	CC	125	47 (19–77)	0/125	I-II	NR	71/54	82/97.8	78/95.3	7
Wang, Y. C., et al. (2011)	China	Osteosarcoma	76	NR	25/51	NR	37	36/40	33.8/67.1	24.9/56.3	6
Yamashita, H., et al. (2006)	Japan	BC	506	NR (22–91)	0/506	NR	NR	206/300	83.2/74.7	72.7/65.3	7
Zhang, L. J., et al. (2013)	China	Wilms’ tumor	58	31 (3–132)	38/20	I-IV	≥ 78	17/41	31.4/74.1	31.4/74.1	7

M1: Marker1, STAT3; M2: Marker 2, p-STAT3; N: nuclear expression; C: cytoplasmic expression; 1: Cohort 1; 2: Cohort 2; NSCLC: Non-Small Cell Lung Cancer; GC: Gastric cancer; CRC: Colorectal Cancer; BC: Breast cancer; CC: Cervical Carcinoma; OC: Ovarian Carcinoma; PDA: Pancreatic Ductal Adenocarcinoma; TET: Thymic Epithelial Tumours; RCC: Renal Cell Carcinoma; HC: Hepatocellular Carcinoma; HNSCC: Head and Neck Squamous Cell Carcinoma; EC: Esophageal Cancer; OSCC: Oral Squamous Cell Carcinoma; SCLC: Small Cell Lung Cancer; NC: Nasopharyngeal carcinoma; LSCC: Lingual Squamous Cell Carcinoma; PC: Prostate Cancer; NR: Not Reported; DFS: disease-free survival, STAT3: Signal transducer and activator of transcription protein 3; NOS: newcastle–Ottawa Scale; OS: overall survival.

### Evaluation and expression of STAT3

Antibodies, detection and definition method, and cut-off values of STAT3 expression used in the included studies is summarized in Table 2. Diverse antibodies were used for the assessment of STAT3 expression by IHC. For anti-STAT3 antibody, three studies used clone sc-8019, one study each used clone RB-9237, F-2, sc-7179, 79D7, 124H6, and sixteen studies did not report the antibody clone. For anti-p-STAT3 antibody, eight studies used clone D3A7, four studies used clone sc-7993, two studies used clone 9131, one study each used sc-483, sc-8001, sc-8059, ZP-0647, and twenty studies did not report the antibody clone. The median expression of STAT3 in solid tumors was 47.79%, range from 19.65% to 94.66%.

**Table 2:** Evaluation of human STAT3/p-STAT3 by IHC in the selected studies

References	Type of cancer	Marker	Cutoff	Antibody (Clone)
Abou-Ghazal, M., et al. (2008)	Gliomas	p-STAT3	NR	anti-p-STAT3 (Tyr705), Cell Signaling Technology
Ai, T., et al. (2012)	NSCLC	STAT3	IHC > 51%	anti-STAT3, Cell Signaling Technology
Birner, P., etal. (2010)	Gliomas	p-STAT3	IHC ≥ 5%	anti-p-STAT3 (Tyr705), clone D3A7, Cell Signaling
Chang, K. C., et al. (2006)	TET	STAT3	IHC ≥ 10%	anti-Stat3 F-2: sc-8019, Santa Cruz Biotechnology, Inc.
Chatterjee, Devasis., et al. (2008)	GC	STAT3-nuclear	IHC scores ≥ 4	anti-STAT3, Santa Cruz Biotechnology, Inc.
Chen, H. H., et al. (2012)	CC	STAT3	IHC ≥ 20%	anti-STAT3, Santa Cruz Biotechnology, Inc.
Chen, C. C., et al. (2010)	NC	p-STAT3	IHC > 10%	NR
Choi, Chel Hun., et al. (2010)	CC	p-STAT3	IHC > 51%	anti-p-STAT3 (ser727), Santa Cruz Biotechnology
Cortas, T., et al. (2007)	NSCLC	p-STAT3	IHC ≥ 5%	anti-p-STAT3 (sc-8059), Santa Cruz Biotechnology
Deng, J. Y., et al. (2010)	GC	p-STAT3	≥ 10%	anti-p-STAT3 (sc-483)
Deng, J., et al. (2013)	GC	p-STAT3	IHC > 25%	anti-p-STAT3, Santa, sc-8001-R
Denley, S. M., et al. (2013)	PDA	p-STAT3	IHC ≥ 2%	anti-pStat3 Tyr 705, 9131, Cell Signaling Technology
				anti-pStat3 (Ser 727), 9134, Cell Signaling Technology
Dobi, E., et al. (2013)	CRC	p-STAT3	IHC > 15%	anti-p-STAT3, sc-7993, Santa Cruz Biotechnology
Dolled-Filhart, M., et al. (2003)	BC	STAT3-cytoplasmic	IHC score ≥ 1	anti-STAT3, Cell Signaling Technology
		STAT3-nuclear		anti-STAT3, Cell Signaling Technology
		p-STAT3-cytoplasmic		anti-p-STAT3 (Tyr 705), Cell Signaling Technology
		p-STAT3-nuclear		anti-p-STAT3 (Tyr 705), Cell Signaling Technology
Galleges Ruiz, M. I., et al. (2009)	NSCLC	p-STAT3-nuclear	IHC score > 210	anti–p-STAT3
Gordziel, C., et al. (2013)	CRC	STAT3-cytoplasmic	IHC score ≥ 2	anti-STAT3: Stat3 (79D7), Cell Signaling Technology
		STAT3-nuclear		
Haura, Eric B., et al. (2005)	NSCLC	p-STAT3-nuclear	IHC score ≥ 1	anti-p-Stat3 (Tyr 705), Cell Signaling Technology
Hbibi, A. Tadlaoui., et al. (2008)	CRC	p-STAT3	IHC score ≥ 6	anti-P-STAT3 (Tyr 705), Cell Signaling Technology
		STAT3		anti-STAT3, Cell Signaling
Horiguchi, Akio., et al. (2002)	RCC	p-STAT3	IHC ≥ 10%	anti-p-STAT3, (Tyr 705), Cell Signaling Technology
Huang, C., et al. (2012)	PDA	p-STAT3	IHC ≥ 25%	anti-p-STAT3, Cell Signaling Technology
Jia, Yanfei., et al. (2013)	GC	STAT3	NR	anti-STAT3, Santa Cruz Biotechnology
Kim, D. Y., et al. (2009)	GC	STAT3	NR	anti-STAT3, Chemicon International
Kim, Yeon-Joo., et al. (2011)	NC	STAT3	IHC ≥ 10%	anti-STAT3, Epitomics
Kusaba, T., et al. (2006)	CRC	p-STAT3	IHC > 15%	anti-p-STAT3 (Tyr705), Santa Cruz Biotechnology
Lee, I., et al. (2012)	Melanoma	p-STAT3	IHC ≥ 1%	anti-p-STAT3 (Tyr705), Santa Cruz Biotechnology
Lee, J., et al. (2009)	GC	p-STAT3	IHC ≥ 1%	anti-p-STAT3 (Tyr705), Cell Signaling Technology
Li, Chao., et al. (2013)	TET	STAT3	IHC > 10%	anti-STAT3, Santa Cruz Biotechnology
Li, X., et al. (2015)	NSCLC	STAT3	IHC score ≥ 4	anti-STAT3, Santa Cruz Biotechnology
Lin, G. S., et al. (2014)	Gliomas	p-STAT3	IHC > 5%	anti-p-STAT3 (Tyr705), D3A7, Cell Signaling
Macha, Muzafar A., et al. (2011)	Oral cancer	p-STAT3	NR	anti-p-STAT3 (Tyr 705), Cell Signaling
Mano, Y., et al. (2013)	HC	p-STAT3	NR	anti-p-STAT3 (Tyr 705), D3A7, Cell Signaling
Min, Hao., et al. (2009)	OC	STAT3	IHC ≥ 10%	anti-Stat3, (SC-8019), Santa Cruz Biotechnology
		p-STAT3	IHC ≥ 10%	anti-p-Stat3 (Tyr 705), ZP-0647, Abzoom Biotechnology
Monnien, F., et al. (2010)	CRC	p-STAT3	IHC > 15%	anti-p-Stat3 (Tyr 705), sc-7993, Santa Cruz
Pectasides, Eirini., et al. (2010)	HNSCC	STAT3-nuclear	NR	anti-Stat3, clone 124H6; Cell Signaling Technology
Piperi, Christina., et al. (2011)	Gliomas	p-STAT3	IHC ≥ 6%	anti-p-STAT3 (Tyr 705), D3A7 XP, Cell Signaling
Rosen, D. G., et al. (2006)	OC	p-STAT3	IHC > 10%	anti-p-Stat3, (SC-7993-R), Santa Cruz Biotechnology
Ryu, Keinosuke., et al. (2010)	Osteosarcoma	p-STAT3	IHC > 51%	anti-p-STAT, Cell Signaling Technology
Schoppmann, Sebastian F., et al. (2012)	EC	p-STAT3	IHC > 10%	anti-p-STAT3 (Tyr 705), D3A7, Cell Signaling
Shah, N. G., et al. (2006)	OSCC	STAT3-nuclear	IHC > 10%	anti-STAT3, Santa Cruz Biotechnology
Slinger, E., et al. (2010)	Gliomas	p-STAT3	IHC > 30%	anti-p-STAT3, (Tyr 705), Cell Signaling
Sheen-Chen, Shyr-Ming., et al. (2008)	BC	STAT3	IHC score ≥ 3	anti-STAT3 (RB-9237), NeoMarkers
Sonnenblick, A., et al. (2012)	BC	p-STAT3	IHC ≥ 25%	anti-p-STAT3, (Tyr 705), Cell Signaling
Sonnenblick, A., et al. (2013)	BC	p-STAT3	IHC ≥ 10%	anti-p-STAT3, (Tyr 705), Cell Signaling
Takemoto, S., et al. (2009)	CC	p-STAT3	IHC ≥ 5%	anti-p-Stat3 (Tyr 705), sc-7993, Santa Cruz Biotechnology
Tam, L., et al. (2007)	PC	p-STAT3-cytoplasmic	ICCC > 0.7	anti-p-STAT3 (Tyr 705), 9131, Cell Signaling
		p-STAT3-nuclear		
van Cruijsen, H., et al. (2009)	NSCLC	p-STAT3	NR	anti-p-STAT3 (Tyr 705), clone D3A7, Cell Signaling
Wang, M., et al. (2011)	NSCLC	p-STAT3	IHC > 25%	anti-p-STAT3, Cell Signaling Technology
Wang, Y., et al. (2011)	Gliomas	p-STAT3	IHC score > 4	anti-p-STAT3 (Tyr 705), clone D3A7, Cell Signaling
Wang, Y. C., et al. (2011)	Osteosarcoma	STAT3	IHC > 5%	anti-STAT3, Santa Cruz Biotechnology
Wu, Zheng-Sheng., et al. (2011)	Melanoma	p-STAT3	NR	anti-p-STAT3, Santa Cruz Biotechnology
Woo, S., et al. (2011)	GC	p-STAT3	IHC ≥ 1%	anti-p-STAT3, (Tyr 705), Cell Signaling
Xiong, Hua., et al. (2012)	GC	STAT3	IHC > 15%	anti-STAT3
		p-STAT3		anti-p-STAT3 (Tyr 705)
Yakata, Yuichi., et al. (2007)	GC	p-STAT3	IHC > 10%	anti-p-STAT3, Santa Cruz Biotechnology
Yamashita, H., et al. (2006)	BC	STAT3	IHC score ≥ 2	anti-STAT3, (F-2), Santa Cruz Biotechnology
Yang, C., et al. (2013)	OC	p-STAT3	IHC > 50%	anti-p-STAT3, (Tyr 705), Cell Signaling Technology
Yang, Cao., et al. (2009)	Chordoma	p-STAT3	IHC score ≥ 4	anti-p-STAT3, Cell Signaling Technology
Yin, Z., et al. (2012)	NSCLC	STAT3	IHC ≥ 50%	anti-STAT3, (sc-8019); Santa Cruz
You, Z., et al. (2012)	EC	p-STAT3	IHC score ≥ 2	anti-p-STAT3, (Tyr 705), Cell Signaling Technology
Yu, Y., et al. (2015)	NSCLC	pSTAT3	IHC score ≥ 3	NR
Zhang, C. H., et al. (2012)	HC	STAT3	IHC > 10%	anti-STAT3, Santa Cruz Biotechnology
		p-STAT3		anti-p-STAT3, (Tyr 705), Cell Signaling Technology
Zhang, L. J., et al. (2013)	Wilms' tumor	STAT3	IHC > 51%	anti-STAT3, (sc-7179), Santa Cruz Biotechnology
Zhao, X., et al. (2012)	SCLC	STAT3	IHC ≥ 25%	anti-STAT3, Wuhan Boster Company
		p-STAT3		anti-p-STAT3, clone B-7, Wuhan Boster Company
Zhao, Yan., et al. (2012)	LSCC	STAT3	IHC ≥ 10%	anti-STAT3, Santa Cruz Biotechnology

NSCLC: Non-Small Cell Lung Cancer; GC: Gastric cancer; CRC: Colorectal Cancer; BC: Breast cancer; CC: Cervical Carcinoma; OC: Ovarian Carcinoma; PDA: Pancreatic Ductal Adenocarcinoma; TET: Thymic Epithelial Tumours; RCC: Renal Cell Carcinoma; HC: Hepatocellular Carcinoma; HNSCC: Head and Neck Squamous Cell Carcinoma; EC: Esophageal Cancer; OSCC: Oral Squamous Cell Carcinoma; SCLC: Small Cell Lung Cancer; NC: Nasopharyngeal carcinoma; LSCC: Lingual Squamous Cell Carcinoma; PC: Prostate Cancer; ICCH: Interclass Correlation Coefficient; NR: Not Reported.

### Association of STAT3 with OS

**Figure 2:**
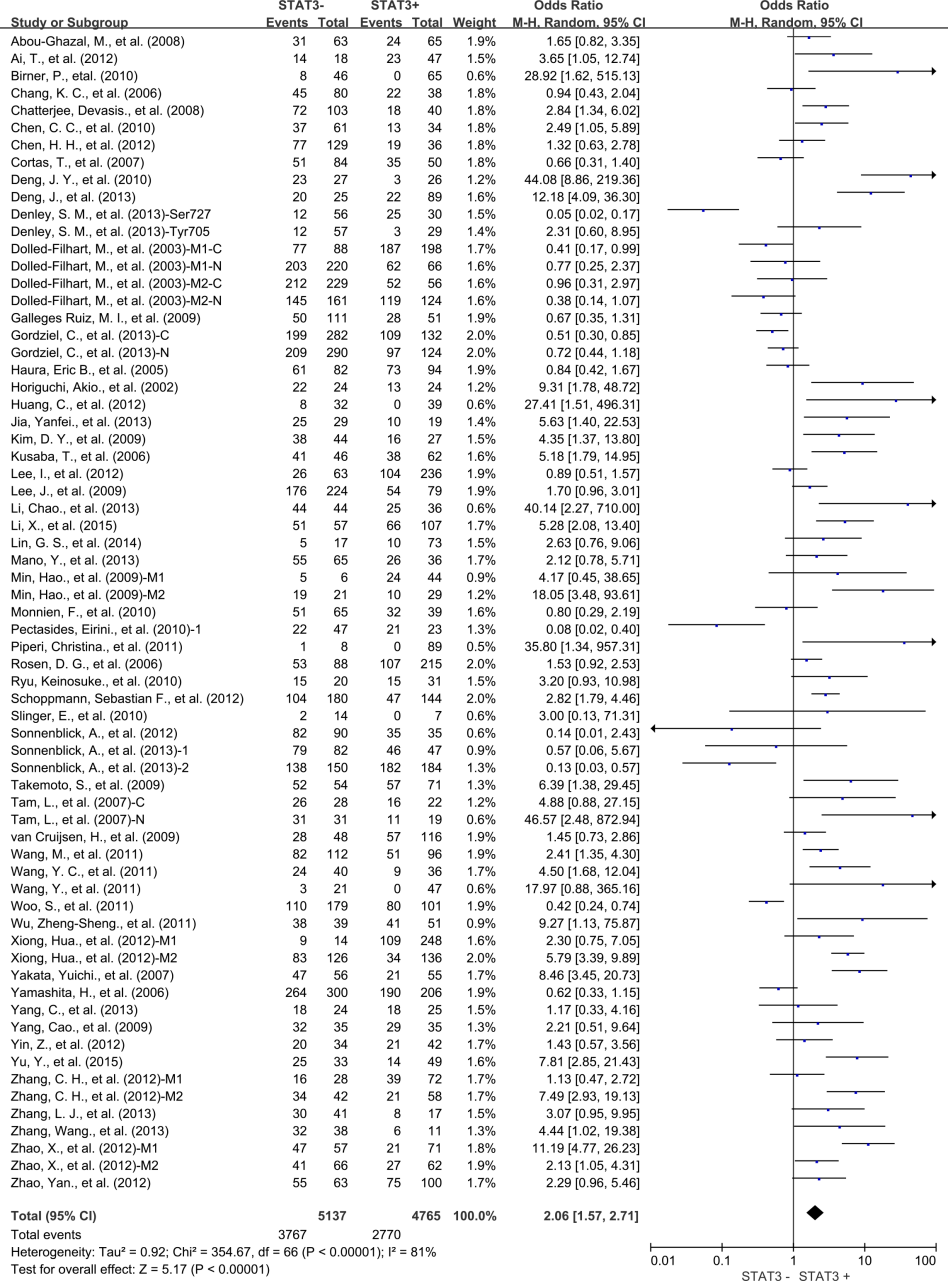
Three-year overall survival (OS) by STAT3 expression M1: Marker 1, STAT3; M2: Marker 2, p-STAT3; 1: Cohort 1; 2: Cohort 2; N: nuclear expression; C: cytoplasmic expression.

The combined analysis of 54 studies showed that STAT3 overexpression in tumor tissue was associated with worse 3-year OS of solid tumors (OR = 2.06, 95% CI = 1.57 to 2.71, *P* < 0.00001) (Figure 2). There was significant heterogeneity among studies (Cochran's Q *P* < 0.00001, I^2^ = 81%), so we conducted meta-regression analysis and subgroup meta-analysis to investigate the possible source of the heterogeneity among studies.

**Figure 3:**
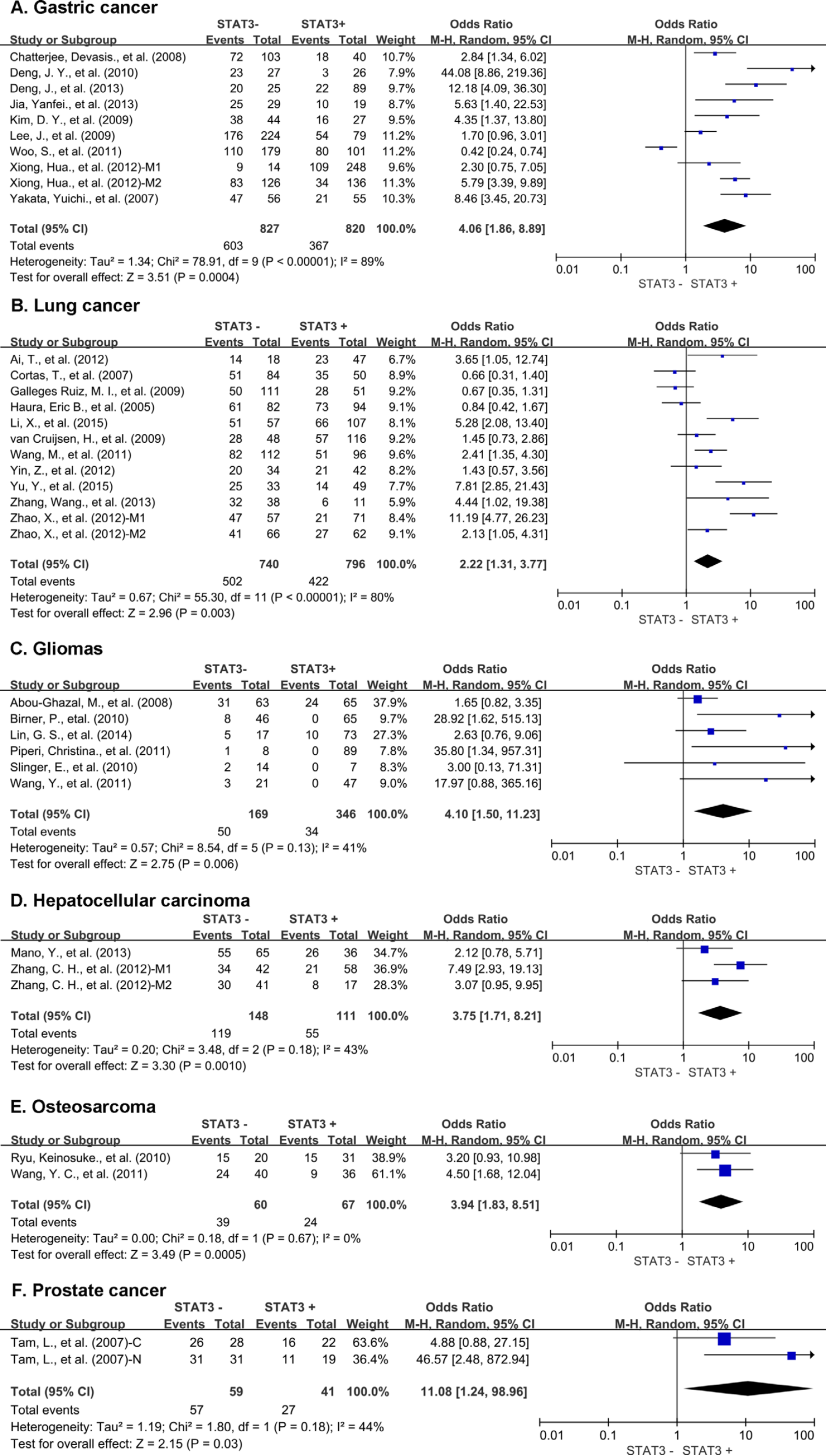
Subgroup analysis of 3-year OS by STAT3 expression in different tumor types (**A**) gastric cancer; (**B**) lung cancer; (**C**) gliomas; (**D**) hepatic cancer; (**E**) osteosarcoma; (**F**) prostate cancer. M1: Marker 1, STAT3; M2: Marker 2, p-STAT3; 1: Cohort 1; 2: Cohort 2 N: nuclear expression; C: cytoplasmic expression.

In the stratified analysis by tumor types, STAT3 expression was associated with worse 3-year OS of gastric cancer (OR = 4.06, 95% CI = 1.86 to 8.89, *P =* 0.0004), lung cancer (OR = 2.22, 95% CI = 1.31 to 3.77, *P =* 0.003), gliomas (OR = 4.10, 95% CI = 1.50 to 11.23, *P =* 0.006), hepatic cancer (OR = 3.75, 95% CI = 1.71 to 8.21, *P =* 0.001), osteosarcoma (OR = 3.94, 95% CI = 1.83 to 8.51, *P =* 0.0005) and prostate cancer (OR = 11.08, 95% CI = 1.24 to 98.96, *P =* 0.03) (Figure 3). There was no significant association between STAT3 expression and 3-year OS of colorectal cancer, ovarian cancer, pancreatic cancer, cervical cancer, melanoma and thymic epithelial tumor (Supplementary Figure S1). Interestingly, STAT3 overexpression was associated with favorable 3-year OS of breast cancer (OR = 0.51, 95% CI = 0.35 to 0.74, *P =* 0.0004) (Supplementary Figure S2).

Meta-regression analysis showed that publication year, country, gender and NOS score did not contribute to the heterogeneity (data not shown).

**Figure 4:**
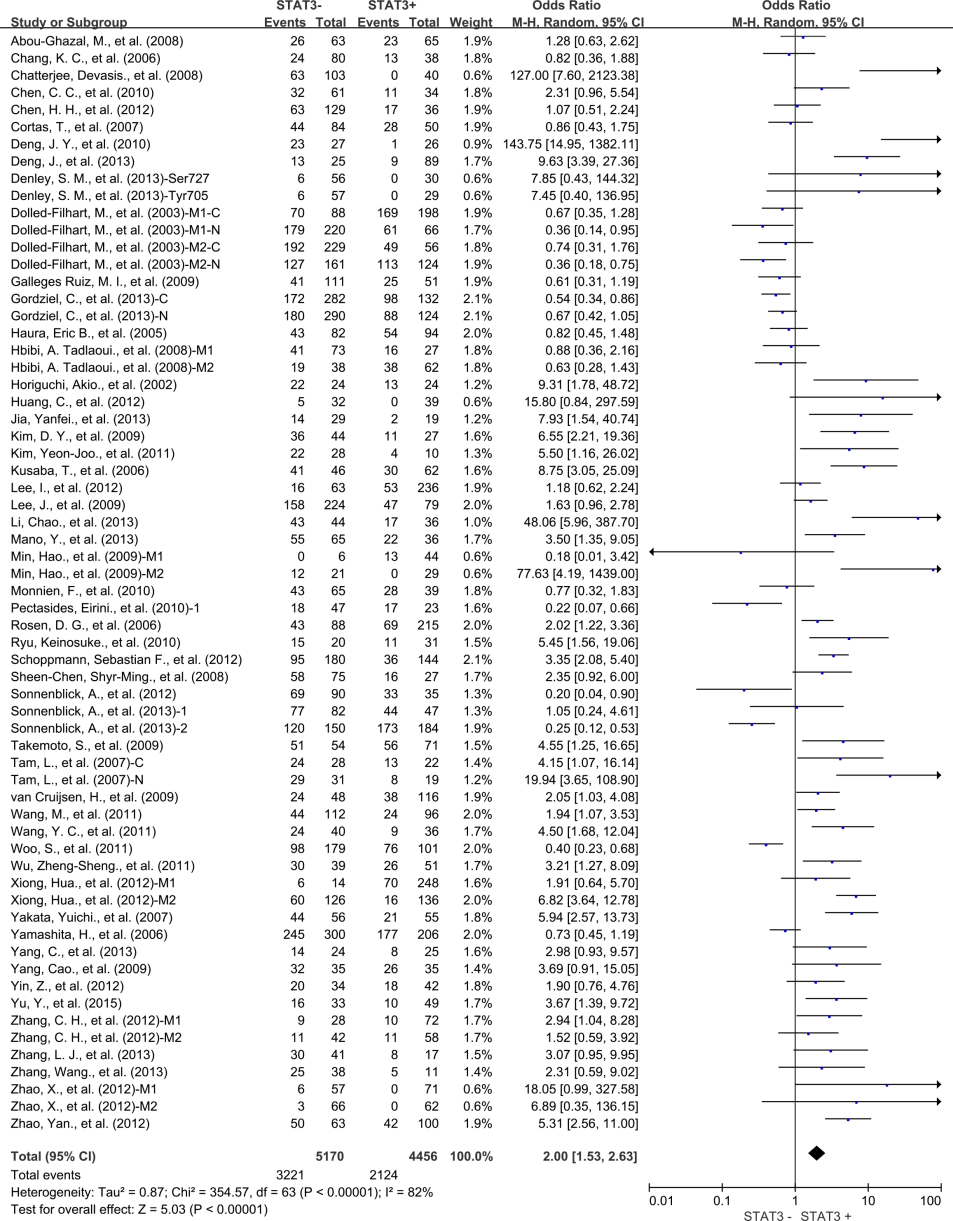
Five-year OS by STAT3 expression M1: Marker 1, STAT3; M2: Marker 2, p-STAT3; 1: Cohort 1; 2: Cohort 2 N: nuclear expression; C: cytoplasmic expression.

Analysis of 49 studies showed STAT3 expression was also associated with worse 5-year OS (OR = 2.00, 95% CI = 1.53 to 2.63, *P* < 0.00001) (Figure 4) of solid tumors. There was also high heterogeneity among studies for 5-year OS (Cochran's Q *P* < 0.00001, I^2^ = 82%), so we conducted subgroup meta-analysis.

**Figure 5:**
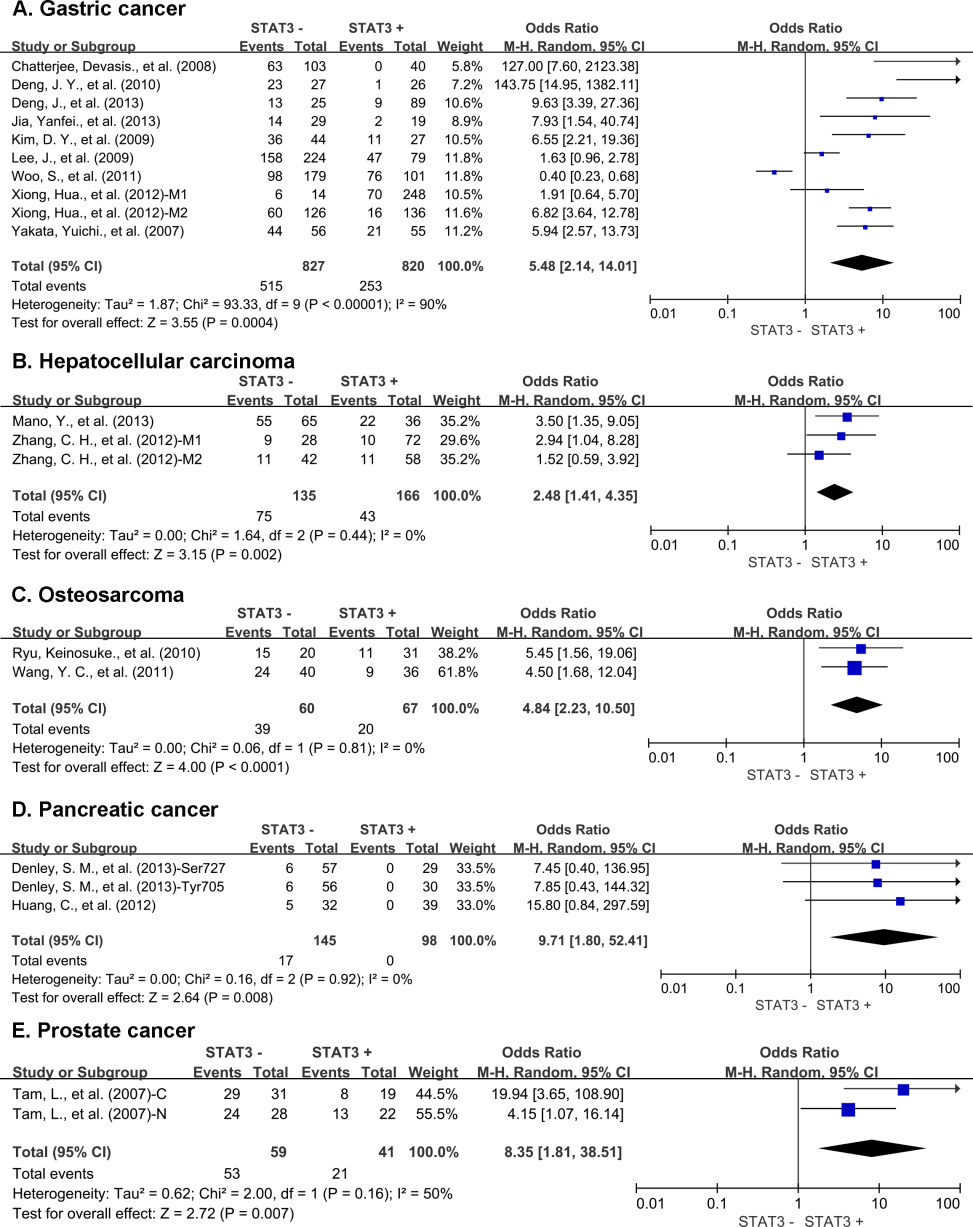
Subgroup analysis of 5-year OS by STAT3 expression in different tumor types (**A**) gastric cancer; (**B**) hepatic cancer; (**C**) osteosarcoma; (**D**) pancreatic cancer; (**E**) prostate cancer. M1: Marker 1, STAT3; M2: Marker 2, p-STAT3; 1: Cohort 1; 2: Cohort 2 N: nuclear expression; C: cytoplasmic expression.

Subgroup analysis showed that STAT3 expression was associated with worse 5-year OS of gastric cancer (OR = 5.48, 95% CI = 2.14 to 14.01, *P =* 0.0004), hepatic cancer (OR = 2.48, 95% CI = 1.41 to 4.35, *P =* 0.002), osteosarcoma (OR = 4.84, 95% CI = 2.23 to 10.50, *P* < 0.0001), pancreatic cancer (OR = 9.71, 95% CI = 1.80 to 52.41, *P =* 0.008) and prostate cancer (OR = 8.35, 95% CI = 1.81 to38.51, *P =* 0.007) (Figure 5). There was no significant association between STAT3 expression and the 5-year OS of colorectal cancer, lung cancer, ovarian cancer, cervical cancer, melanoma and thymic epithelial tumor (Supplementary Figure S3). STAT3 overexpression was associated with favorable 5-year OS of breast cancer (OR = 0.57, 95% CI = 0.37 to 0.89, *P =* 0.01) (Supplementary Figure S4).

Twenty studies evaluated STAT3, 38 studies evaluated p-STAT3 and 5 studies evaluated both STAT3 and p-STAT3. Our result showed that both STAT3 and p-STAT3 overexpression were associated with worse OS of solid tumors. However, elevated p-STAT3 (OR = 2.45, 95% CI = 1.73 to 3.46, *P* < 0.00001) expression in tumor tissue seemed to be more significantly associated with worse 3-year OS than STAT3 expression (OR = 1.72, 95% CI = 1.10 to 2.70, *P =* 0.02) (Supplementary Figure S5). Similar result was observed for 5-year OS analysis (Supplementary Figure S6). A subgroup meta-analysis of studies evaluated both STAT3 and p-STAT3 shown that p-STAT3 expression was associated with worse 3-year and 5-year OS of solid tumor, but not STAT3 (Supplementary Figure S7).

**Figure 6:**
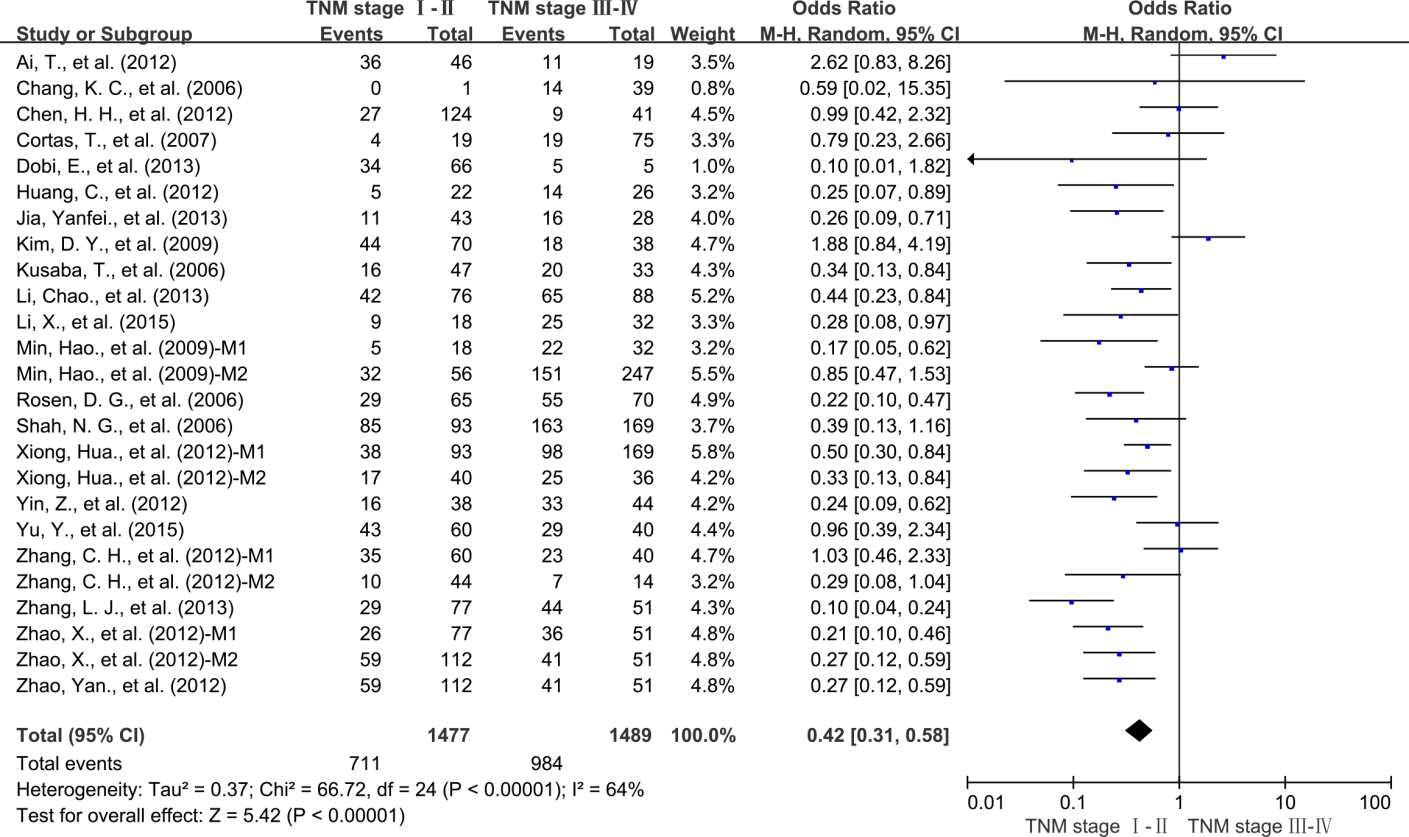
Subgroup analysis the correlation of STAT3 expression and tumor stage M1: Marker 1, STAT3; M2: Marker 2, p-STAT3.

We also evaluated the correlation between STAT3 overexpression and the TNM stage of tumor. High expression level of STAT3 was significantly associated with advanced TNM stage (OR = 0.42, 95% CI = 0.31 to 0.58, *P* < 0.00001) (Figure 6).

**Figure 7:**
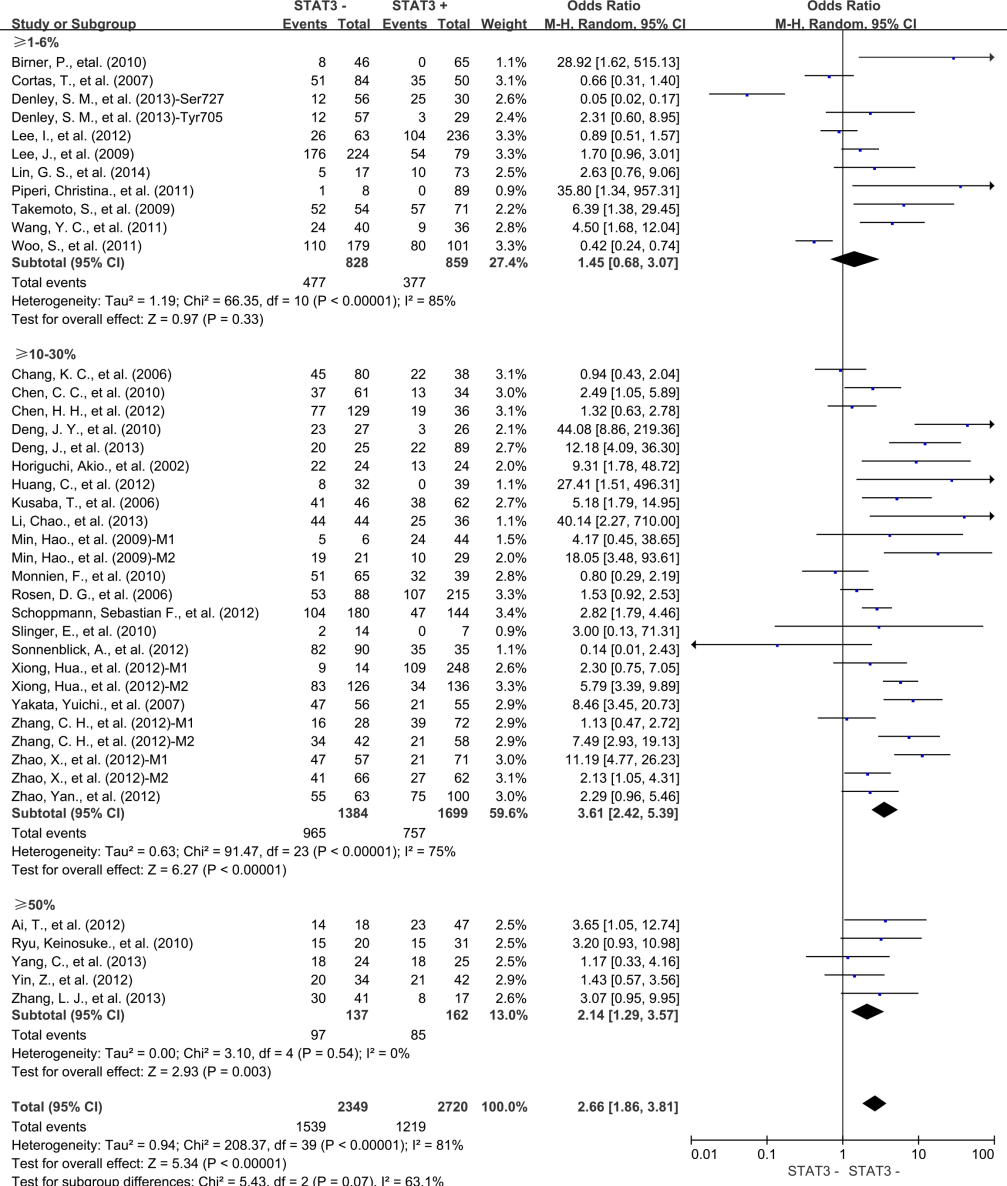
Subgroup analysis the correlation between STAT3 overexpression and 3-year OS of solid tumors according to cut-off values determining STAT3 positivity M1: Marker 1, STAT3; M2: Marker 2, p-STAT3.

Next, we conducted subgroup analysis according to STAT3 expression level. Results showed STAT3 expression was associated with poor 3-year OS in the studies using cutoff values of 10%–30% (OR = 3.61, 95% CI = 2.42 to 5.39, *P* < 0.00001) and 50% (OR = 2.14, 95% CI = 1.29 to 3.57, *P =* 0.003) (Figure 7) to determine STAT3 positivity. Similar result was observed in 5-year OS (Supplementary Figure S6). However, the studies used cutoff value of STAT3 overexpression as more than 1%-6% tumor cells positive was not associated with 3-year and 5-year OS of solid tumors.

### Association of STAT3 with DFS

**Figure 8:**
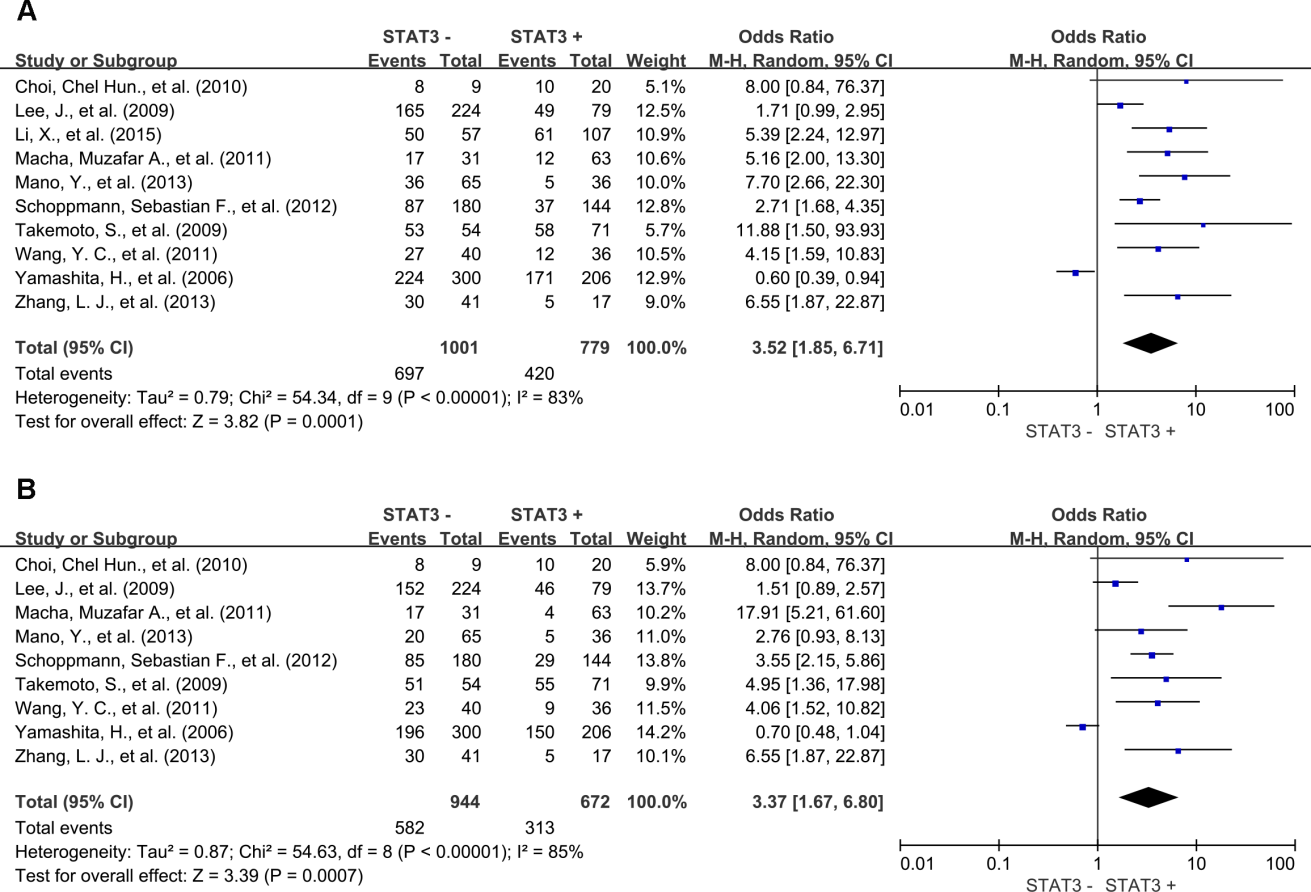
Three and five-year DFS by STAT3 expression (**A**) 3-year DFS; (**B**) 5-year DFS. M1: Marker 1, STAT3; M2: Marker 2, p-STAT3.

Meta-analysis of 10 studies showed that STAT3 expression was associated with statistically significant poor 3-year DFS (OR = 3.52, 95% CI = 1.85 to 6.71, *P =* 0.0001) (Figure 8A) and poor 5-year DFS (OR = 3.37, 95% CI = 1.67 to 6.80, *P =* 0.0007) (Figure 8B).

### Sensitivity analyses

Removal of the studies that was an outlier (score, IRS, > 50% vs 1%–6% for other studies) or no report (NR) with regard to the cutoff of STAT3 overexpression by IHC did not influence results for 3- or 5-year OS (OR = 2.45, 95% CI = 1.73 to 3.48, *p* < 0.00001; OR = 2.08, 95% CI = 1.46 to 2.96, *p* < 0.0001; respectively). Exclusion of these studies did not reduce heterogeneity for 3- or 5-year OS (Cochran's Q *P* < 0.00001, I^2^ = 82%; Cochran's Q *P* < 0.00001, I^2^ = 84%, respectively).

Removal of studies with NOS score 6 did not influence results for 3- or 5-year OS (OR = 2.49, 95% CI = 1.86 to 3.34, *p* < 0.00001; OR = 2.33, 95% CI = 1.74 to 3.12, *p* < 0.00001, respectively). Exclusion of these studies did not reduce heterogeneity for 3- or 5-year OS (Cochran's Q *P* < 0.00001, I^2^ = 80%; Cochran's Q *P* < 0.00001, I^2^ = 82%, respectively).

### Publication bias

Funnel plot analysis showed that there was no statistical evidence of publication bias in our meta-analysis (data not shown).

## DISCUSSION

This meta-analysis is the most comprehensive assessment of the literatures regarding STAT3 expression and tumor prognosis to date. We systematically evaluated survival data for 9449 solid tumor patients included in 63 different studies. Our study demonstrated that the expression of STAT3 is a marker of poor prognosis in solid tumors, with consistent results of OS at 3 and 5 years. Regarding to the tumor types, elevated STAT3 expression in tumor tissues were associated with worse OS of gastric cancer, lung cancer, gliomas, hepatic cancer, osteosarcoma, prostate cancer and pancreatic cancer. However, elevated STAT3 expression was associated with better prognosis of breast cancer. In addition, expression level of phosphorylated STAT3 was more significantly associated with worse outcome of solid tumors than unphosphorylated STAT3.

Our study found there is no significant correlation between STAT3 overexpression and OS of colorectal cancer and ovarian cancer. And STAT3 overexpression in breast cancer tissue is associated with favorable OS. However, recent studies demonstrated that STAT3-targeted inhibitor could restrain tumor development in various solid tumor models including breast cancer [16, 19, 85, 86], melanoma [87] and ovarian cancer [16, 88]. These divergences suggest that further study is needed to shed more light on the underling mechanism of STAT3 signal pathway in pro-tumor microenvironment in different tumor types.

There are several important implications in this meta-analysis. First, it shows that STAT3 expression is related to adverse outcome of most solid tumors. Second, it identifies a subgroup of tumors with unfavorable outcome in gastric cancer, lung cancer, hepatic cancer, prostate cancer and glioblastoma, but with favorable outcome in breast cancer. Finally, it emphasizes the potential of STAT3 to developing a valuable therapeutic target and prognostic biomarker for solid tumor.

This study also has some limitations. First, from the literature we could only extract summarized population-level data rather than individual patient-level data. Second, the method for assessing STAT3 expression and definition of STAT3 positivity are inconsistent. Finally, substantial heterogeneity observed across included studies cannot be fully accounted for by our use of appropriate meta-analytic techniques with random-effects modeling.

In summary, STAT3 expression in solid tumor tissues is associated with poor survival in most solid tumors, which suggests that STAT3 is a valuable prognostic biomarker and a promising therapeutic target for solid tumors.

## MATERIALS AND METHODS

This meta-analysis was conducted according to the statement for reporting systematic reviews and meta-analyses [89]. This study summarized and analyzed the results of previous studies, so the ethical approval was not necessary.

### Search strategy and study selection

An electronic search of Pubmed, Web of Science and EBSCO were undertaken for studies evaluating STAT3 or p-STAT3 expression and clinical outcome in solid tumors from 1994 to August 2015. The search was performed with subject heading terms including “signal transducer and activator of transcription 3” or “STAT3 transcription factor” or “STAT3” or “phosphorylated signal transducer and activator of transcription 3” or “phosphorylated STAT3 transcription factor” or “phospho-STAT3” and “neoplasms” and the results were limited to human studies of solid tumors. In addition, the entry “signal transducer and activator of transcription 3” or “STAT3 transcription factor” or “STAT3” or “phosphorylated signal transducer and activator of transcription 3” or “phosphorylated STAT3 transcription factor” or “phospho-STAT3” and the name of each specific solid tumor were used for additional studies. A total of 3547, 3542 and 2914 entries were identified, respectively. Inclusion criteria were the measurement of STAT3 and (or) p-STAT3 by immunohistochemistry (IHC), availability of survival data for at least 3 years, and original article written in English. Exclusion criteria were studies evaluating gene expression of STAT3 measured by polymerase chain reaction (PCR) and STAT3 expression in lymph node and myeloid cells. Citation lists of retrieved articles were manually screened to ensure sensitivity of the search strategy. Study selection was based on the association of STAT3 and survival. Two reviewers (Pin Wu and Dang Wu) evaluated independently all of the full articles for study eligibility. Inter-reviewer agreement was assessed using Cohen's kappa coefficient. Disagreement was resolved by consensus.

### Data extraction

Overall survival (OS) and disease free survival (DFS) were the primary endpoints of interest. Data were extracted using predefined abstraction forms. The following details were extracted by two authors (Pin Wu and Dang Wu): name of first author, year of publication, country of publication, tumor type, patient number, tumor stage, antibodies used for the evaluation, method and score for STAT3 assessment, and cut-off values to determine STAT3 positivity. Data for 3 and 5 year of OS and DFS were extracted from tables or Kaplan–Meier curves for both STAT3 negative and STAT3 positive group.

The studies included in our meta-analysis were all cohort studies. Two independent authors evaluated the quality of each included study using Newcastle-Ottawa Scale (NOS) [90]. The studies with 6 scores or more were considered as high quality studies. A consensus NOS score for each item was achieved finally.

### Data synthesis

The relative frequency of OS and DFS at 3 and 5 years between STAT3 negative and STAT3 positive group was presented as an odds ratio (OR) and its 95% confidence interval (CI). Sensitivity analyses were carried out for different analytical methods and cut-offs for defining STAT3 expression and NOS scores for quality assessment of included studies. Publication bias was assessed by visual inspection of the funnel plot.

### Statistical analysis

Data were extracted from the primary publications and combined into a meta-analysis using RevMan 5.3 analysis software (Cochrane Collaboration, Copenhagen, Denmark). Estimates of ORs were weighted and pooled using the Mantel–Haenszel random effect model. Statistical heterogeneity was assessed using the Cochran's Q and I^2^ statistics. Differences between subgroups were assessed using methods as previous described by Deeks et al. [91]. Meta-regression analysis was conducted using Stata 12.0 software (StataCorp LP, College Station, TX). All statistical tests were two-sided, and statistical significance was defined as P less than 0.05. No correction was made for multiple statistical testing.

## SUPPLEMENTARY MATERIALS FIGURES


