# Supplementary material for: Prognostic role of STAT3 in solid tumors: a systematic review and meta-analysis
Source: Oncotarget. 2016 Mar 3;7(15):19863–83. doi: 10.18632/oncotarget.7887 (PMC4991424; doi:10.18632/oncotarget.7887)
Supplement: Supplementary file 1 [file oncotarget-07-19863-s001.pdf]

Prognostic role of STAT3 in solid tumors: a systematic review and meta-analysis

Supplementary Materials

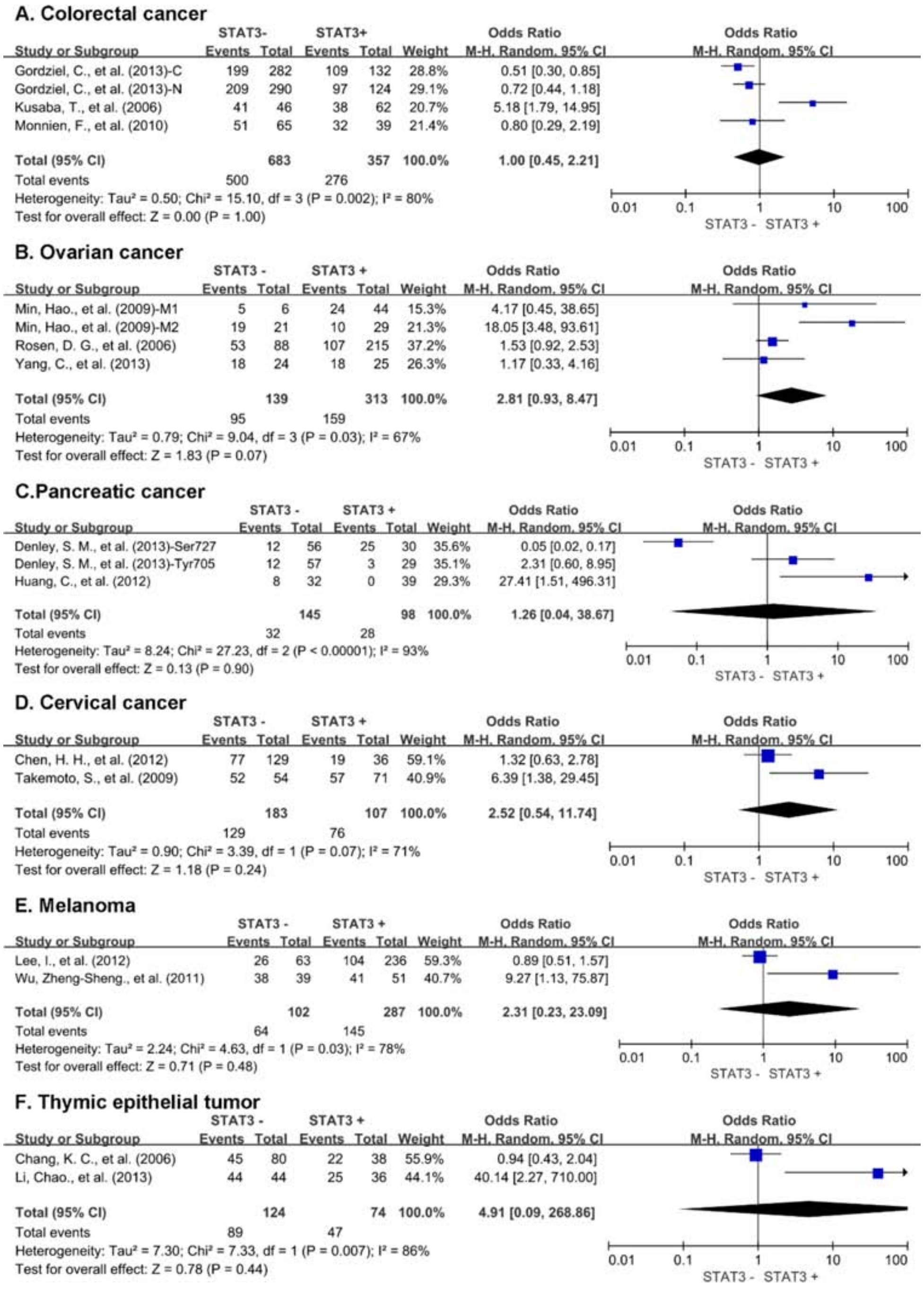

Supplementary Figure S1: Subgroup analysis of 3-year OS by STAT3 expression in different tumor types. (**A**) colorectal cancer; (**B**) ovarian cancer; (**C**) pancreatic cancer; (**D**) cervical cancer; (**E**) melanoma; (**F**) thymic epithelial tumor. M1: Marker 1, STAT3; M2: Marker 2, p-STAT3; N: nuclear expression; C: cytoplasmic expression.

Study or Subgroup	STAT3- Events	STAT3- Total	STAT3+ Events	STAT3+ Total	Weight	Odds Ratio M-H, Random, 95% CI
Dolled-Filhart, M., et al. (2003)-M1-C	77	88	187	198	18.1%	0.41 [0.17, 0.99]
Dolled-Filhart, M., et al. (2003)-M1-N	203	220	62	66	11.0%	0.77 [0.25, 2.37]
Dolled-Filhart, M., et al. (2003)-M2-C	212	229	52	56	10.9%	0.96 [0.31, 2.97]
Dolled-Filhart, M., et al. (2003)-M2-N	145	161	119	124	13.1%	0.38 [0.14, 1.07]
Sonnenblick, A., et al. (2012)	82	90	35	35	1.7%	0.14 [0.01, 2.43]
Sonnenblick, A., et al. (2013)-1	79	82	46	47	2.7%	0.57 [0.06, 5.67]
Sonnenblick, A., et al. (2013)-2	138	150	182	184	6.1%	0.13 [0.03, 0.57]
Yamashita, H., et al. (2006)	264	300	190	206	36.5%	0.62 [0.33, 1.15]
**Total (95% CI)**		**1320**		**916**	**100.0%**	**0.51 [0.35, 0.74]**
Total events	1200		873			

Heterogeneity: Tau² = 0.00; Chi² = 6.78, df = 7 (P = 0.45); I² = 0%
Test for overall effect: Z = 3.52 (P = 0.0004)

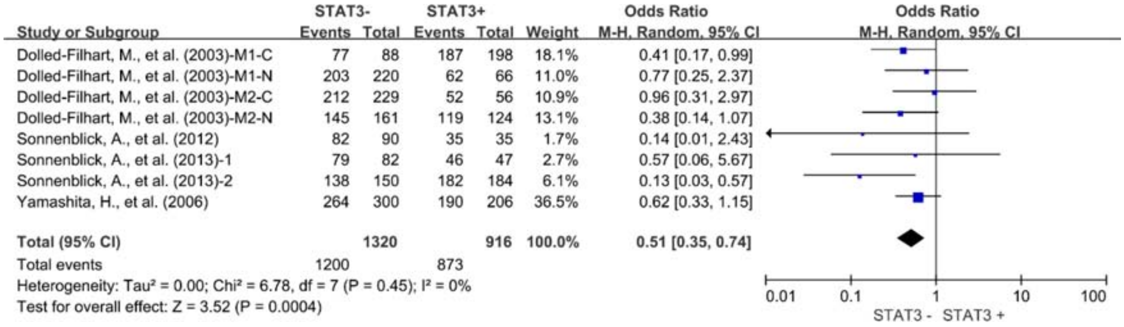

Supplementary Figure S2: Subgroup analysis of 3-year OS by STAT3 expression in breast cancer. M1: Marker 1, STAT3; M2: Marker 2, p-STAT3; N: nuclear expression; C: cytoplasmic expression.

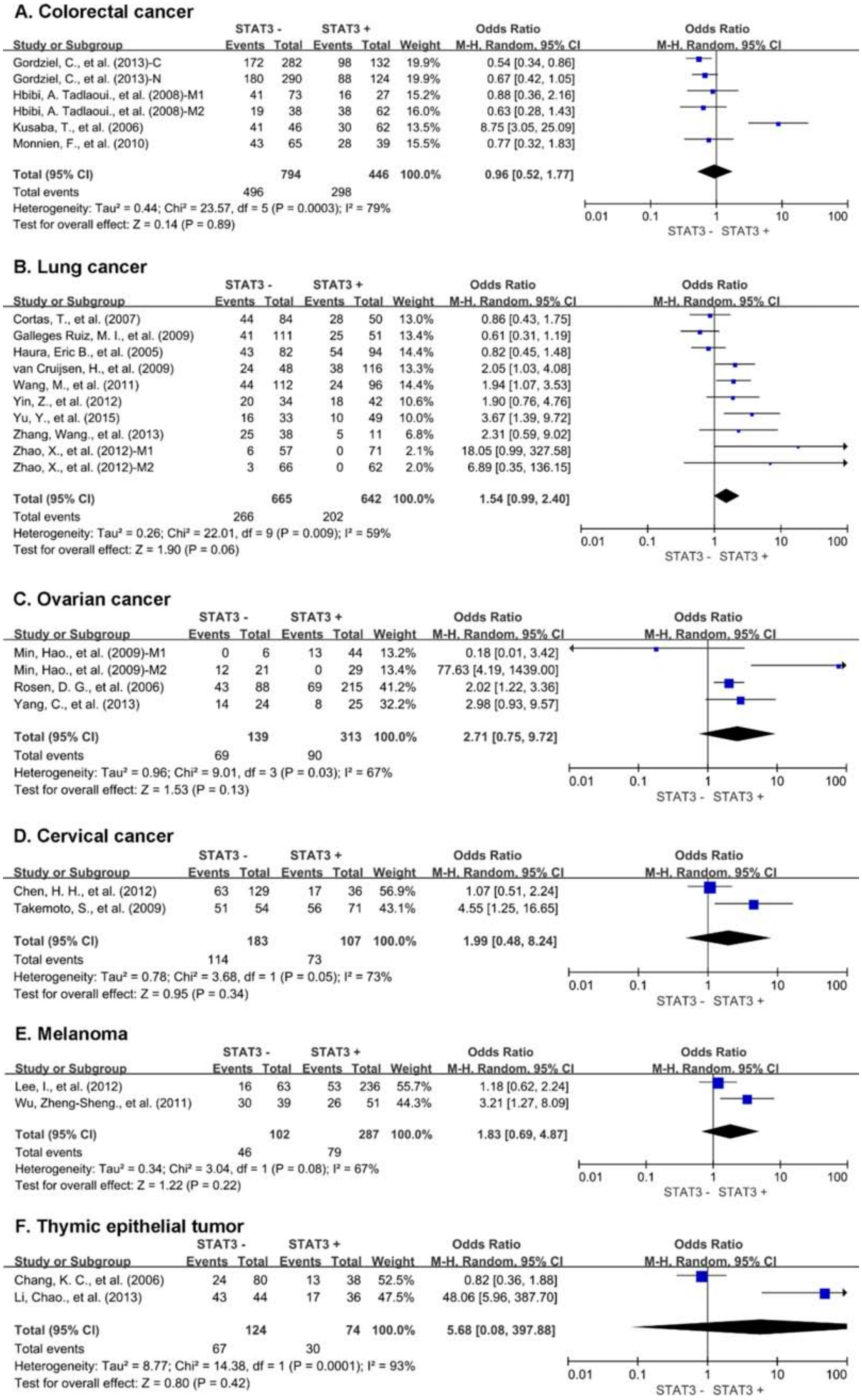

Supplementary Figure S3: Subgroup analysis of 5-year OS by STAT3 expression in different tumor types. (**A**) colorectal cancer; (**B**) lung cancer; (**C**) ovarian cancer; (**D**) cervical cancer; (**E**) melanoma; (**F**) thymic epithelial tumor. M1: Marker 1, STAT3; M2: Marker 2, p-STAT3; N: nuclear expression; C: cytoplasmic expression.

Study or Subgroup	STAT3 - Events	STAT3 - Total	STAT3 + Events	STAT3 + Total	Weight	Odds Ratio M-H, Random, 95% CI
Dolled-Filhart, M., et al. (2003)-M1-C	70	88	169	198	14.0%	0.67 [0.35, 1.28]
Dolled-Filhart, M., et al. (2003)-M1-N	179	220	61	66	10.1%	0.36 [0.14, 0.95]
Dolled-Filhart, M., et al. (2003)-M2-C	192	229	49	56	11.3%	0.74 [0.31, 1.76]
Dolled-Filhart, M., et al. (2003)-M2-N	127	161	113	124	13.0%	0.36 [0.18, 0.75]
Sheen-Chen, Shyr-Ming., et al. (2008)	58	75	16	27	10.5%	2.35 [0.92, 6.00]
Sonnenblick, A., et al. (2012)	69	90	33	35	6.0%	0.20 [0.04, 0.90]
Sonnenblick, A., et al. (2013)-1	77	82	44	47	6.1%	1.05 [0.24, 4.61]
Sonnenblick, A., et al. (2013)-2	120	150	173	184	12.9%	0.25 [0.12, 0.53]
Yamashita, H., et al. (2006)	245	300	177	206	16.1%	0.73 [0.45, 1.19]
**Total (95% CI)**		**1395**		**943**	**100.0%**	**0.57 [0.37, 0.89]**
Total events	1137		835			

Heterogeneity: $Tau^2 = 0.25$; $Chi^2 = 19.92$, df = 8 (P = 0.01); $I^2 = 60\%$

Test for overall effect: Z = 2.48 (P = 0.01)

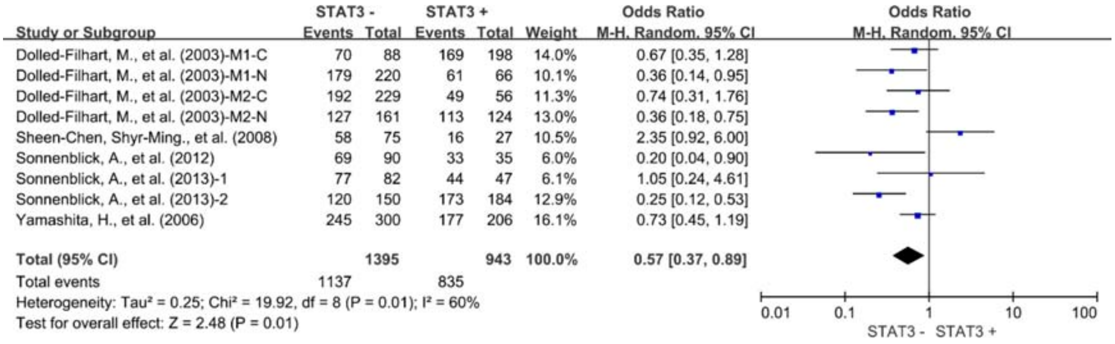

Supplementary Figure S4: Subgroup analysis of 5-year OS by STAT3 expression in breast cancer. M1: Marker 1, STAT3; M2: Marker 2, p-STAT3; N: nuclear expression; C: cytoplasmic expression.

A. p-STAT3

Study or Subgroup	p-STAT3 - Events	p-STAT3 - Total	p-STAT3 + Events	p-STAT3 + Total	Weight	Odds Ratio M-H, Random, 95% CI
Abou-Ghazal, M., et al. (2008)	31	63	24	65	3.0%	1.65 [0.82, 3.35]
Birner, P., etal. (2010)	8	46	0	65	1.0%	28.92 [1.62, 515.13]
Chen, C. C., et al. (2010)	37	61	13	34	2.8%	2.49 [1.05, 5.89]
Cortas, T., et al. (2007)	51	84	35	50	2.9%	0.66 [0.31, 1.40]
Deng, J. Y., et al. (2010)	23	27	3	26	2.0%	44.08 [8.86, 219.36]
Deng, J., et al. (2013)	20	25	22	89	2.5%	12.18 [4.09, 36.30]
Denley, S. M., et al. (2013)-Ser727	12	56	25	30	2.5%	0.05 [0.02, 0.17]
Denley, S. M., et al. (2013)-Tyr705	12	57	3	29	2.2%	2.31 [0.60, 8.95]
Dolled-Filhart, M., et al. (2003)-M2-C	212	229	52	56	2.5%	0.96 [0.31, 2.97]
Dolled-Filhart, M., et al. (2003)-M2-N	145	161	119	124	2.6%	0.38 [0.14, 1.07]
Galleges Ruiz, M. I., et al. (2009)	50	111	28	51	3.0%	0.67 [0.35, 1.31]
Haura, Eric B., et al. (2005)	61	82	73	94	3.0%	0.84 [0.42, 1.67]
Horiguchi, Akio., et al. (2002)	22	24	13	24	1.9%	9.31 [1.78, 48.72]
Huang, C., et al. (2012)	8	32	0	39	1.0%	27.41 [1.51, 496.31]
Kusaba, T., et al. (2006)	41	46	38	62	2.6%	5.18 [1.79, 14.95]
Lee, I., et al. (2012)	26	63	104	236	3.1%	0.89 [0.51, 1.57]
Lee, J., et al. (2009)	176	224	54	79	3.1%	1.70 [0.96, 3.01]
Lin, G. S., et al. (2014)	5	17	10	73	2.4%	2.63 [0.76, 9.06]
Mano, Y., et al. (2013)	55	65	26	36	2.7%	2.12 [0.78, 5.71]
Min, Hao., et al. (2009)-M2	19	21	10	29	1.9%	18.05 [3.48, 93.61]
Monnien, F., et al. (2010)	51	65	32	39	2.6%	0.80 [0.29, 2.19]
Piperi, Christina., et al. (2011)	1	8	0	89	0.8%	35.80 [1.34, 957.31]
Rosen, D. G., et al. (2006)	53	88	107	215	3.2%	1.53 [0.92, 2.53]
Ryu, Keinosuke., et al. (2010)	15	20	15	31	2.4%	3.20 [0.93, 10.98]
Schoppmann, Sebastian F., et al. (2012)	104	180	47	144	3.2%	2.82 [1.79, 4.46]
Slinger, E., et al. (2010)	2	14	0	7	0.9%	3.00 [0.13, 71.31]
Sonnenblick, A., et al. (2012)	82	90	35	35	1.0%	0.14 [0.01, 2.43]
Takemoto, S., et al. (2009)	52	54	57	71	2.0%	6.39 [1.38, 29.45]
Tam, L., et al. (2007)-C	26	28	16	22	1.8%	4.88 [0.88, 27.15]
Tam, L., et al. (2007)-N	31	31	11	19	1.0%	46.57 [2.48, 872.94]
van Cruijsen, H., et al. (2009)	28	48	57	116	3.0%	1.45 [0.73, 2.86]
Wang, M., et al. (2011)	82	112	51	96	3.1%	2.41 [1.35, 4.30]
Wang, Y., et al. (2011)	3	21	0	47	0.9%	17.97 [0.88, 365.16]
Woo, S., et al. (2011)	110	179	80	101	3.1%	0.42 [0.24, 0.74]
Wu, Zheng-Sheng., et al. (2011)	38	39	41	51	1.5%	9.27 [1.13, 75.87]
Xiong, Hua., et al. (2012)-M2	83	126	34	136	3.1%	5.79 [3.39, 9.89]
Yakata, Yuichi., et al. (2007)	47	56	21	55	2.8%	8.46 [3.45, 20.73]
Yang, C., et al. (2013)	18	24	18	25	2.3%	1.17 [0.33, 4.16]
Yang, Cao., et al. (2009)	32	35	29	35	2.1%	2.21 [0.51, 9.64]
Yu, Y., et al. (2015)	25	33	14	49	2.6%	7.81 [2.85, 21.43]
Zhang, C. H., et al. (2012)-M2	34	42	21	58	2.7%	7.49 [2.93, 19.13]
Zhang, Wang., et al. (2013)	32	38	6	11	2.1%	4.44 [1.02, 19.38]
Zhao, X., et al. (2012)-M2	41	66	27	62	3.0%	2.13 [1.05, 4.31]
**Total (95% CI)**		**2891**		**2805**	**100.0%**	**2.45 [1.73, 3.46]**
Total events	2004		1371			

Heterogeneity: Tau² = 0.92; Chi² = 223.85, df = 42 (P < 0.00001); I² = 81%
Test for overall effect: Z = 5.07 (P < 0.00001)

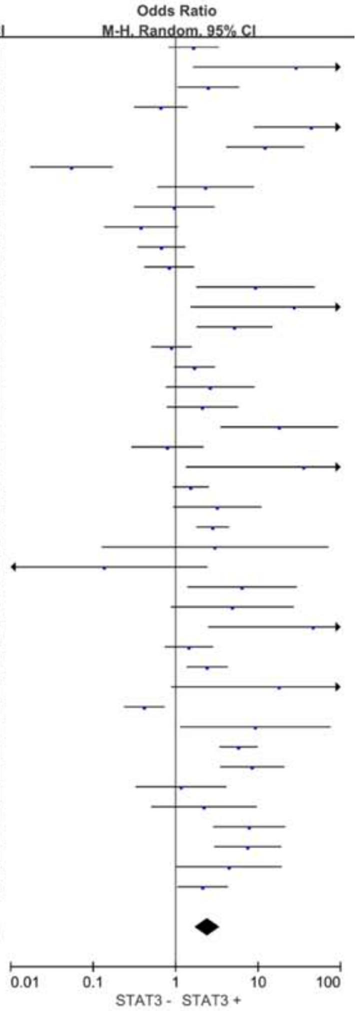

B. STAT3

Study or Subgroup	STAT3 - Events	STAT3 - Total	STAT3 + Events	STAT3 + Total	Weight	Odds Ratio M-H, Random, 95% CI
Ai, T., et al. (2012)	14	18	23	47	4.1%	3.65 [1.05, 12.74]
Chang, K. C., et al. (2006)	45	80	22	38	5.2%	0.94 [0.43, 2.04]
Chatterjee, Devasis., et al. (2008)	72	103	18	40	5.2%	2.84 [1.34, 6.02]
Chen, H. H., et al. (2012)	77	129	19	36	5.2%	1.32 [0.63, 2.78]
Dolled-Filhart, M., et al. (2003)-M1-C	77	88	187	198	5.0%	0.41 [0.17, 0.99]
Dolled-Filhart, M., et al. (2003)-M1-N	203	220	62	66	4.4%	0.77 [0.25, 2.37]
Gordziel, C., et al. (2013)-C	199	282	109	132	5.7%	0.51 [0.30, 0.85]
Gordziel, C., et al. (2013)-N	209	290	97	124	5.7%	0.72 [0.44, 1.18]
Jia, Yanfei., et al. (2013)	25	29	10	19	3.9%	5.63 [1.40, 22.53]
Kim, D. Y., et al. (2009)	38	44	16	27	4.4%	4.35 [1.37, 13.80]
Li, Chao., et al. (2013)	44	44	25	36	1.7%	40.14 [2.27, 710.00]
Li, X., et al. (2015)	51	57	66	107	4.8%	5.28 [2.08, 13.40]
Min, Hao., et al. (2009)-M1	5	6	24	44	2.4%	4.17 [0.45, 38.65]
Pectasides, Eirini., et al. (2010)-1	22	47	21	23	3.5%	0.08 [0.02, 0.40]
Wang, Y. C., et al. (2011)	24	40	9	36	4.7%	4.50 [1.68, 12.04]
Xiong, Hua., et al. (2012)-M1	9	14	109	248	4.4%	2.30 [0.75, 7.05]
Yamashita, H., et al. (2006)	264	300	190	206	5.5%	0.62 [0.33, 1.15]
Yin, Z., et al. (2012)	20	34	21	42	4.9%	1.43 [0.57, 3.56]
Zhang, C. H., et al. (2012)-M1	16	28	39	72	5.0%	1.13 [0.47, 2.72]
Zhang, L. J., et al. (2013)	30	41	8	17	4.3%	3.07 [0.95, 9.95]
Zhao, X., et al. (2012)-M1	47	57	21	71	5.0%	11.19 [4.77, 26.23]
Zhao, Yan., et al. (2012)	55	63	75	100	5.0%	2.29 [0.96, 5.46]
**Total (95% CI)**		**2014**		**1729**	**100.0%**	**1.72 [1.10, 2.70]**
Total events	1546		1171			

Heterogeneity: Tau² = 0.85; Chi² = 109.79, df = 21 (P < 0.00001); I² = 81%
Test for overall effect: Z = 2.39 (P = 0.02)

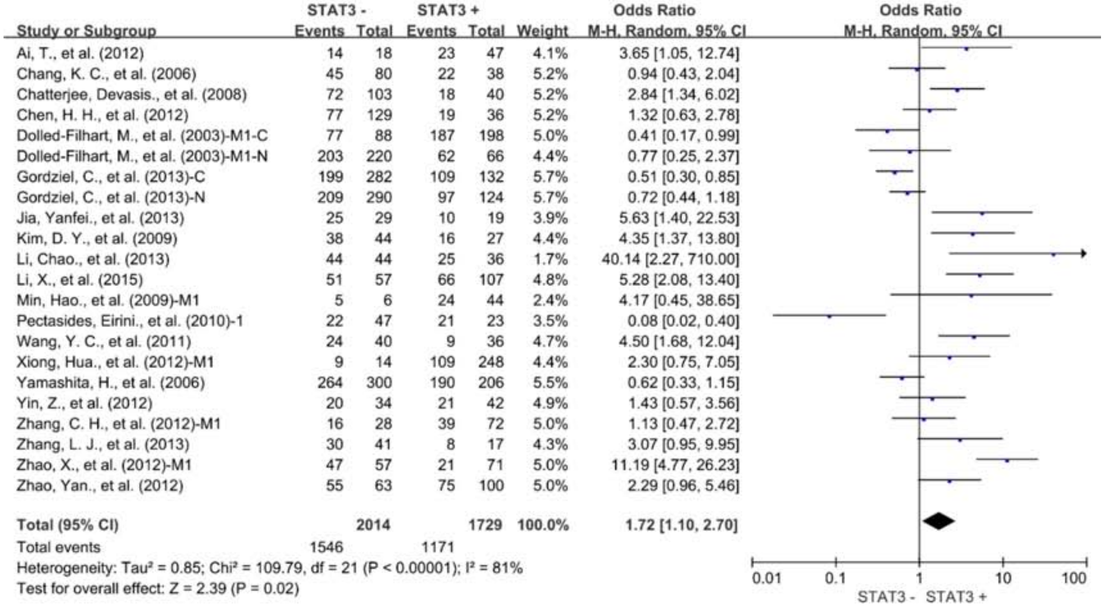

Supplementary Figure S5: Subgroup analysis of 3-year OS according to STAT3 and p-STAT3 expression in solid tumors. (**A**) p-STAT3; (**B**) STAT3; M1: Marker 1, STAT3; M2: Marker 2, p-STAT3; 1: Cohort 1; 2: Cohort 2; N: nuclear expression; C: cytoplasmic expression.

A. p-STAT3

Study or Subgroup	p-STAT3 - Events	p-STAT3 - Total	p-STAT3 + Events	p-STAT3 + Total	Weight	Odds Ratio M-H, Random, 95% CI
Abou-Ghazal, M., et al. (2008)	26	63	23	65	3.2%	1.28 [0.63, 2.62]
Chen, C. C., et al. (2010)	32	61	11	34	2.9%	2.31 [0.96, 5.54]
Cortas, T., et al. (2007)	44	84	28	50	3.2%	0.86 [0.43, 1.75]
Deng, J. Y., et al. (2010)	23	27	1	26	1.4%	143.75 [14.95, 1382.11]
Deng, J., et al. (2013)	13	25	9	89	2.7%	9.63 [3.39, 27.36]
Denley, S. M., et al. (2013)-Ser727	6	56	0	30	1.0%	7.85 [0.43, 144.32]
Denley, S. M., et al. (2013)-Tyr705	6	57	0	29	1.0%	7.45 [0.40, 136.95]
Dolled-Filhart, M., et al. (2003)-M2-C	192	229	49	56	2.9%	0.74 [0.31, 1.76]
Dolled-Filhart, M., et al. (2003)-M2-N	127	161	113	124	3.1%	0.36 [0.18, 0.75]
Galleges Ruiz, M. I., et al. (2009)	41	111	25	51	3.2%	0.61 [0.31, 1.19]
Haura, Eric B., et al. (2005)	43	82	54	94	3.3%	0.82 [0.45, 1.48]
Hbibi, A. Tadlaoui., et al. (2008)-M1	41	73	16	27	2.9%	0.88 [0.36, 2.16]
Horiguchi, Akio., et al. (2002)	22	24	13	24	1.9%	9.31 [1.78, 48.72]
Huang, C., et al. (2012)	5	32	0	39	0.9%	15.80 [0.84, 297.59]
Kusaba, T., et al. (2006)	41	46	30	62	2.7%	8.75 [3.05, 25.09]
Lee, I., et al. (2012)	16	63	53	236	3.2%	1.18 [0.62, 2.24]
Lee, J., et al. (2009)	158	224	47	79	3.4%	1.63 [0.96, 2.78]
Mano, Y., et al. (2013)	55	65	22	36	2.8%	3.50 [1.35, 9.05]
Min, Hao., et al. (2009)-M2	12	21	0	29	1.0%	77.63 [4.19, 1439.00]
Monnien, F., et al. (2010)	43	65	28	39	2.9%	0.77 [0.32, 1.83]
Rosen, D. G., et al. (2006)	43	88	69	215	3.4%	2.02 [1.22, 3.36]
Ryu, Keinosuke., et al. (2010)	15	20	11	31	2.4%	5.45 [1.56, 19.06]
Schoppmann, Sebastian F., et al. (2012)	95	180	36	144	3.4%	3.35 [2.08, 5.40]
Sonnenblick, A., et al. (2012)	69	90	33	35	2.1%	0.20 [0.04, 0.90]
Takemoto, S., et al. (2009)	51	54	56	71	2.4%	4.55 [1.25, 16.65]
Tam, L., et al. (2007)-C	24	28	13	22	2.3%	4.15 [1.07, 16.14]
Tam, L., et al. (2007)-N	29	31	8	19	1.9%	19.94 [3.65, 108.90]
van Cruijsen, H., et al. (2009)	24	48	38	116	3.2%	2.05 [1.03, 4.08]
Wang, M., et al. (2011)	44	112	24	96	3.3%	1.94 [1.07, 3.53]
Woo, S., et al. (2011)	98	179	76	101	3.4%	0.40 [0.23, 0.68]
Wu, Zheng-Sheng., et al. (2011)	30	39	26	51	2.9%	3.21 [1.27, 8.09]
Xiong, Hua., et al. (2012)-M2	60	126	16	136	3.3%	6.82 [3.64, 12.78]
Yakata, Yuichi., et al. (2007)	44	56	21	55	3.0%	5.94 [2.57, 13.73]
Yang, C., et al. (2013)	14	24	8	25	2.5%	2.98 [0.93, 9.57]
Yang, Cao., et al. (2009)	32	35	26	35	2.2%	3.69 [0.91, 15.05]
Yu, Y., et al. (2015)	16	33	10	49	2.8%	3.67 [1.39, 9.72]
Zhang, C. H., et al. (2012)-M2	11	42	11	58	2.8%	1.52 [0.59, 3.92]
Zhang, Wang., et al. (2013)	25	38	5	11	2.3%	2.31 [0.59, 9.02]
Zhao, X., et al. (2012)-M2	3	66	0	62	0.9%	6.89 [0.35, 136.15]
**Total (95% CI)**		**2858**		**2551**	**100.0%**	**2.34 [1.68, 3.25]**
Total events	1673		1009			

Heterogeneity: Tau² = 0.77; Chi² = 192.70, df = 38 (P < 0.00001); I² = 80%

Test for overall effect: Z = 5.02 (P < 0.00001)

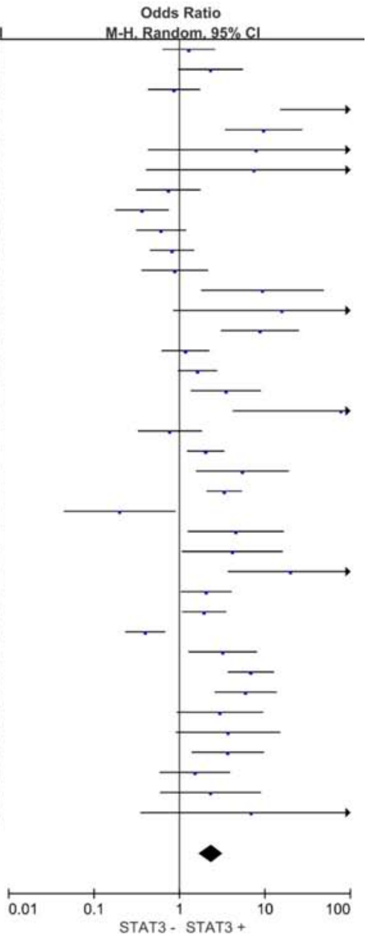

B. STAT3

Study or Subgroup	STAT3 - Events	STAT3 - Total	STAT3 + Events	STAT3 + Total	Weight	Odds Ratio M-H, Random, 95% CI
Chang, K. C., et al. (2006)	24	80	13	38	5.1%	0.82 [0.36, 1.88]
Chatterjee, Devasis., et al. (2008)	63	103	0	40	1.8%	127.00 [7.60, 2123.38]
Chen, H. H., et al. (2012)	63	129	17	36	5.3%	1.07 [0.51, 2.24]
Dolled-Filhart, M., et al. (2003)-M1-C	70	88	169	198	5.4%	0.67 [0.35, 1.28]
Dolled-Filhart, M., et al. (2003)-M1-N	179	220	61	66	4.8%	0.36 [0.14, 0.95]
Gordziel, C., et al. (2013)-C	172	282	98	132	5.8%	0.54 [0.34, 0.86]
Gordziel, C., et al. (2013)-N	180	290	88	124	5.8%	0.67 [0.42, 1.05]
Hbibi, A. Tadlaoui., et al. (2008)-M2	19	38	38	62	5.1%	0.63 [0.28, 1.43]
Jia, Yanfei., et al. (2013)	14	29	2	19	3.4%	7.93 [1.54, 40.74]
Kim, D. Y., et al. (2009)	36	44	11	27	4.5%	6.55 [2.21, 19.36]
Kim, Yeon-Joo., et al. (2011)	22	28	4	10	3.6%	5.50 [1.16, 26.02]
Li, Chao., et al. (2013)	43	44	17	36	2.7%	48.06 [5.96, 387.70]
Min, Hao., et al. (2009)-M1	0	6	13	44	1.7%	0.18 [0.01, 3.42]
Pectasides, Eirini., et al. (2010)-1	18	47	17	23	4.5%	0.22 [0.07, 0.66]
Sheen-Chen, Shyr-Ming., et al. (2008)	58	75	16	27	4.8%	2.35 [0.92, 6.00]
Wang, Y. C., et al. (2011)	24	40	9	36	4.7%	4.50 [1.68, 12.04]
Xiong, Hua., et al. (2012)-M1	6	14	70	248	4.5%	1.91 [0.64, 5.70]
Yamashita, H., et al. (2006)	245	300	177	206	5.7%	0.73 [0.45, 1.19]
Yin, Z., et al. (2012)	20	34	18	42	4.9%	1.90 [0.76, 4.76]
Zhang, C. H., et al. (2012)-M1	9	28	10	72	4.6%	2.94 [1.04, 8.28]
Zhang, L. J., et al. (2013)	30	41	8	17	4.3%	3.07 [0.95, 9.95]
Zhao, X., et al. (2012)-M1	6	57	0	71	1.7%	18.05 [0.99, 327.58]
Zhao, Yan., et al. (2012)	50	63	42	100	5.3%	5.31 [2.56, 11.00]
**Total (95% CI)**		**2080**		**1674**	**100.0%**	**1.72 [1.09, 2.70]**
Total events	1351		898			

Heterogeneity: Tau² = 0.87; Chi² = 122.63, df = 22 (P < 0.00001); I² = 82%

Test for overall effect: Z = 2.35 (P = 0.02)

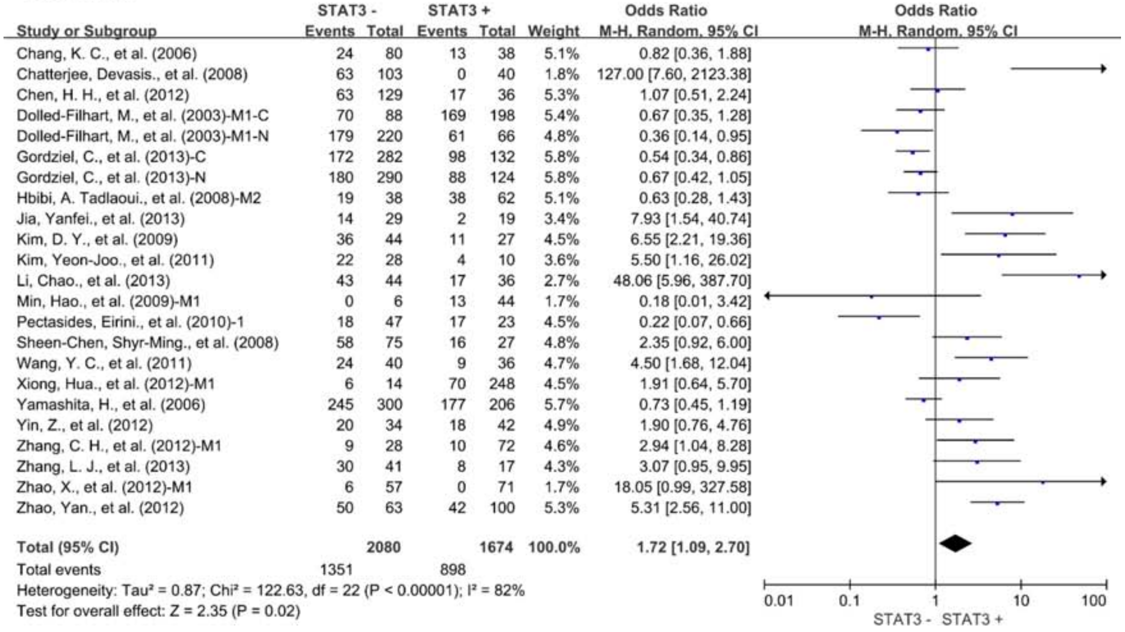

Supplementary Figure S6: Subgroup analysis of 5-year OS according to STAT3 and p-STAT3 expression in solid tumors. (**A**) p-STAT3; (**B**) STAT3; M1: Marker 1, STAT3; M2: Marker 2, p-STAT3; 1: Cohort 1; 2: Cohort 2; N: nuclear expression; C: cytoplasmic expression.

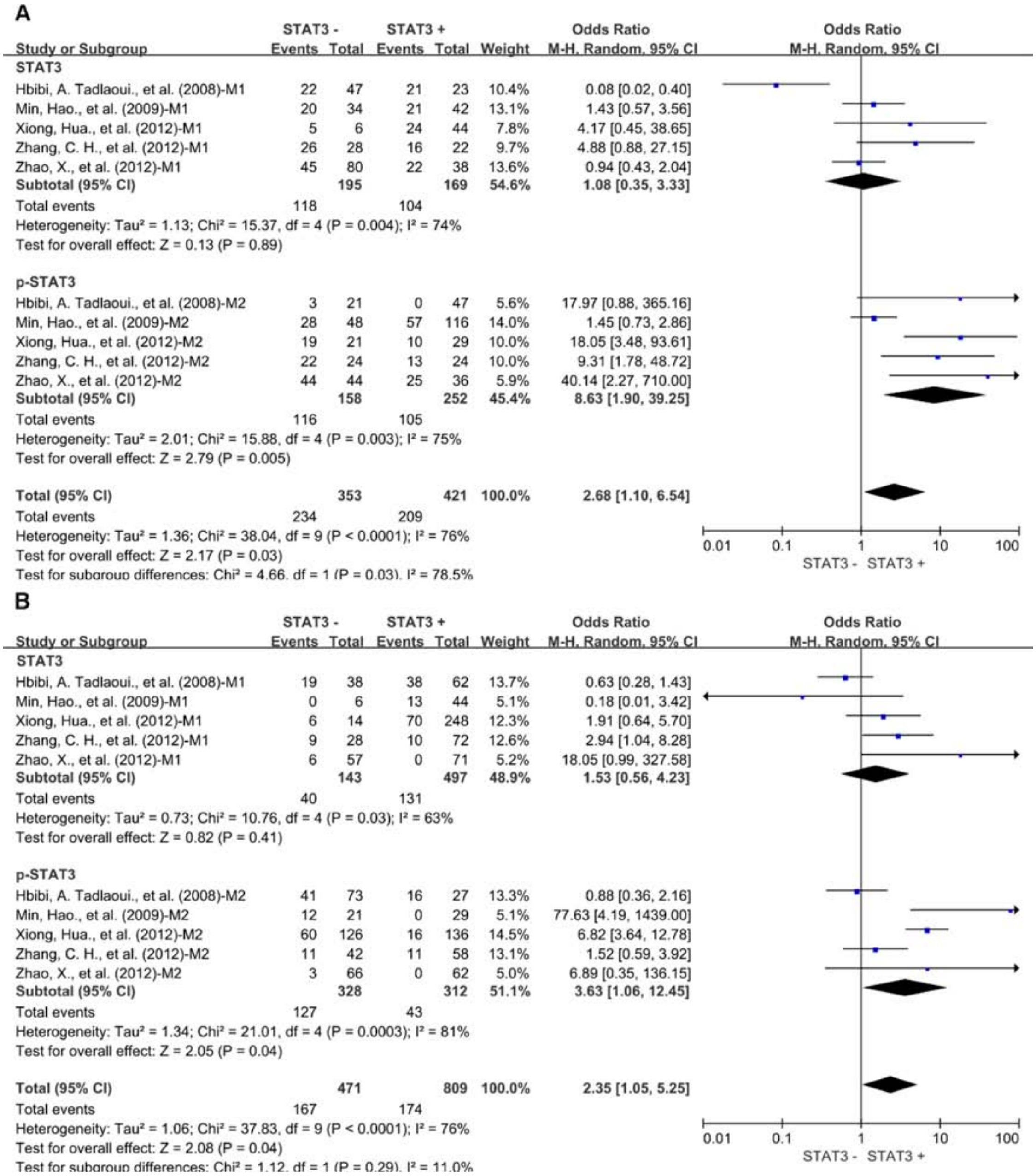

Supplementary Figure S7: Subgroup analysis of OS according to STAT3 and p-STAT3 expression in studies evaluated both STAT3 and p-STAT3. (**A**) 3-year OS; (**B**) 5-year OS; M1: Marker 1, STAT3; M2: Marker 2, p-STAT3; N: nuclear expression; C: cytoplasmic expression.

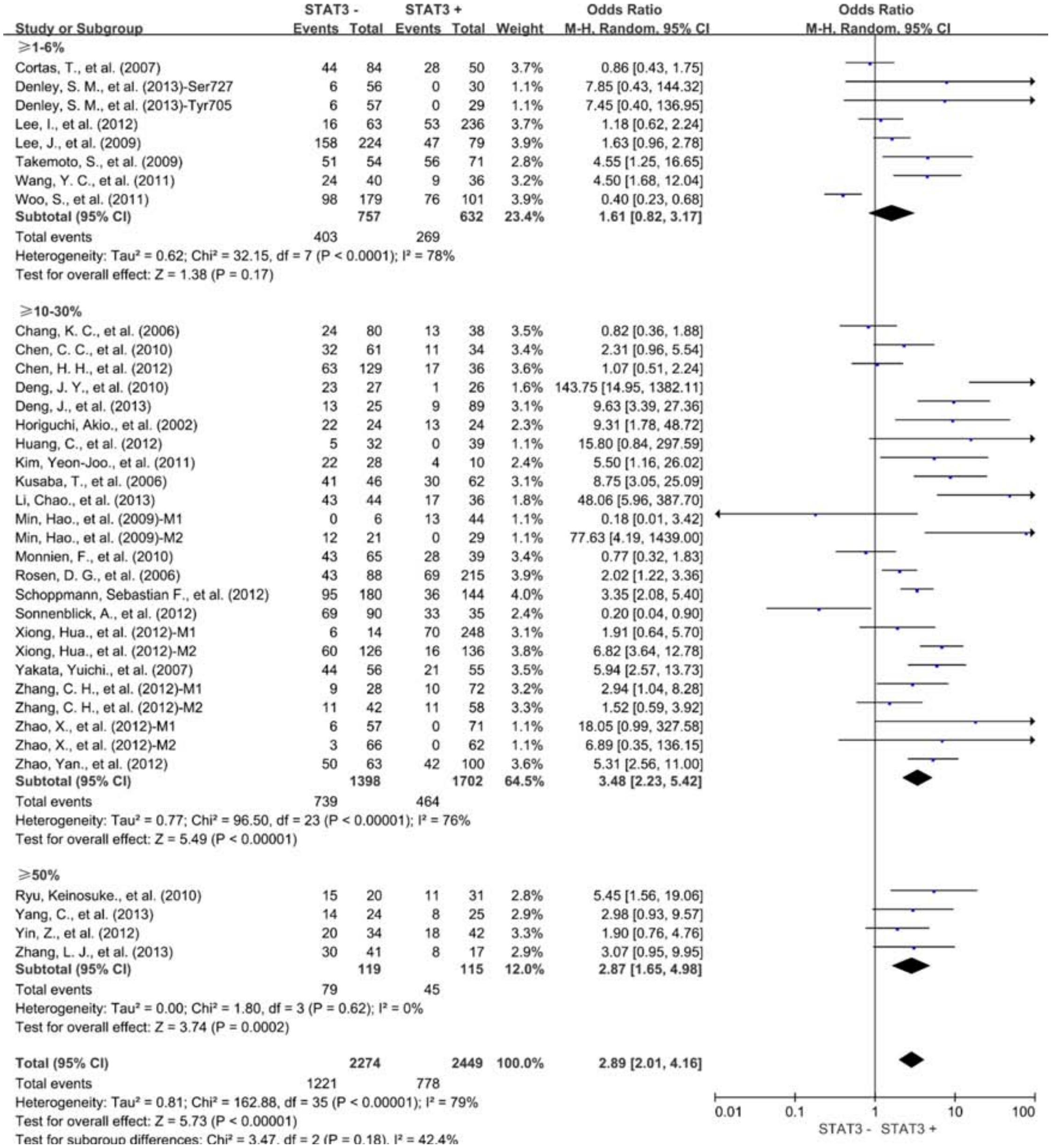

Study or Subgroup	STAT3 - Events	STAT3 - Total	STAT3 + Events	STAT3 + Total	Weight	Odds Ratio M-H, Random, 95% CI
**⩾1-6%**						
Cortas, T., et al. (2007)	44	84	28	50	3.7%	0.86 [0.43, 1.75]
Denley, S. M., et al. (2013)-Ser727	6	56	0	30	1.1%	7.85 [0.43, 144.32]
Denley, S. M., et al. (2013)-Tyr705	6	57	0	29	1.1%	7.45 [0.40, 136.95]
Lee, I., et al. (2012)	16	63	53	236	3.7%	1.18 [0.62, 2.24]
Lee, J., et al. (2009)	158	224	47	79	3.9%	1.63 [0.96, 2.78]
Takemoto, S., et al. (2009)	51	54	56	71	2.8%	4.55 [1.25, 16.65]
Wang, Y. C., et al. (2011)	24	40	9	36	3.2%	4.50 [1.68, 12.04]
Woo, S., et al. (2011)	98	179	76	101	3.9%	0.40 [0.23, 0.68]
**Subtotal (95% CI)**		**757**		**632**	**23.4%**	**1.61 [0.82, 3.17]**
Total events	403		269			
Heterogeneity: Tau² = 0.62; Chi² = 32.15, df = 7 (P < 0.0001); I² = 78%						
Test for overall effect: Z = 1.38 (P = 0.17)						
**⩾10-30%**						
Chang, K. C., et al. (2006)	24	80	13	38	3.5%	0.82 [0.36, 1.88]
Chen, C. C., et al. (2010)	32	61	11	34	3.4%	2.31 [0.96, 5.54]
Chen, H. H., et al. (2012)	63	129	17	36	3.6%	1.07 [0.51, 2.24]
Deng, J. Y., et al. (2010)	23	27	1	26	1.6%	143.75 [14.95, 1382.11]
Deng, J., et al. (2013)	13	25	9	89	3.1%	9.63 [3.39, 27.36]
Horiguchi, Akio., et al. (2002)	22	24	13	24	2.3%	9.31 [1.78, 48.72]
Huang, C., et al. (2012)	5	32	0	39	1.1%	15.80 [0.84, 297.59]
Kim, Yeon-Joo., et al. (2011)	22	28	4	10	2.4%	5.50 [1.16, 26.02]
Kusaba, T., et al. (2006)	41	46	30	62	3.1%	8.75 [3.05, 25.09]
Li, Chao., et al. (2013)	43	44	17	36	1.8%	48.06 [5.96, 387.70]
Min, Hao., et al. (2009)-M1	0	6	13	44	1.1%	0.18 [0.01, 3.42]
Min, Hao., et al. (2009)-M2	12	21	0	29	1.1%	77.63 [4.19, 1439.00]
Monnien, F., et al. (2010)	43	65	28	39	3.4%	0.77 [0.32, 1.83]
Rosen, D. G., et al. (2006)	43	88	69	215	3.9%	2.02 [1.22, 3.36]
Schoppmann, Sebastian F., et al. (2012)	95	180	36	144	4.0%	3.35 [2.08, 5.40]
Sonnenblick, A., et al. (2012)	69	90	33	35	2.4%	0.20 [0.04, 0.90]
Xiong, Hua., et al. (2012)-M1	6	14	70	248	3.1%	1.91 [0.64, 5.70]
Xiong, Hua., et al. (2012)-M2	60	126	16	136	3.8%	6.82 [3.64, 12.78]
Yakata, Yuichi., et al. (2007)	44	56	21	55	3.5%	5.94 [2.57, 13.73]
Zhang, C. H., et al. (2012)-M1	9	28	10	72	3.2%	2.94 [1.04, 8.28]
Zhang, C. H., et al. (2012)-M2	11	42	11	58	3.3%	1.52 [0.59, 3.92]
Zhao, X., et al. (2012)-M1	6	57	0	71	1.1%	18.05 [0.99, 327.58]
Zhao, X., et al. (2012)-M2	3	66	0	62	1.1%	6.89 [0.35, 136.15]
Zhao, Yan., et al. (2012)	50	63	42	100	3.6%	5.31 [2.56, 11.00]
**Subtotal (95% CI)**		**1398**		**1702**	**64.5%**	**3.48 [2.23, 5.42]**
Total events	739		464			
Heterogeneity: Tau² = 0.77; Chi² = 96.50, df = 23 (P < 0.00001); I² = 76%						
Test for overall effect: Z = 5.49 (P < 0.00001)						
**⩾50%**						
Ryu, Keinosuke., et al. (2010)	15	20	11	31	2.8%	5.45 [1.56, 19.06]
Yang, C., et al. (2013)	14	24	8	25	2.9%	2.98 [0.93, 9.57]
Yin, Z., et al. (2012)	20	34	18	42	3.3%	1.90 [0.76, 4.76]
Zhang, L. J., et al. (2013)	30	41	8	17	2.9%	3.07 [0.95, 9.95]
**Subtotal (95% CI)**		**119**		**115**	**12.0%**	**2.87 [1.65, 4.98]**
Total events	79		45			
Heterogeneity: Tau² = 0.00; Chi² = 1.80, df = 3 (P = 0.62); I² = 0%						
Test for overall effect: Z = 3.74 (P = 0.0002)						
**Total (95% CI)**		**2274**		**2449**	**100.0%**	**2.89 [2.01, 4.16]**
Total events	1221		778			
Heterogeneity: Tau² = 0.81; Chi² = 162.88, df = 35 (P < 0.00001); I² = 79%						
Test for overall effect: Z = 5.73 (P < 0.00001)						
Test for subgroup differences: Chi² = 3.47, df = 2 (P = 0.18), I² = 42.4%						

Supplementary Figure S8: Subgroup analysis the correlation between STAT3 overexpression and 5-year OS according to cut-off values determining STAT3 positivity. M1: Marker 1, STAT3; M2: Marker 2, p-STAT3.
